# Distribution, richness and conservation of the genus *Salvia* (Lamiaceae) in the State of Michoacán, Mexico

**DOI:** 10.3897/BDJ.8.e56827

**Published:** 2020-10-29

**Authors:** Mayra Flores-Tolentino, Sabina I. Lara-Cabrera, José Luis Villaseñor

**Affiliations:** 1 Universidad Nacional Autónoma de México, Ciudad de México, Mexico Universidad Nacional Autónoma de México Ciudad de México Mexico; 2 Universidad Michoacana de San Nicolás de Hidalgo, Morelia, Mexico Universidad Michoacana de San Nicolás de Hidalgo Morelia Mexico

**Keywords:** conservation, Maxent, еcological niche models, regularisation, species richness

## Abstract

Little attention has been paid in Mexico to species’ geographical distribution, particularly documenting geographic ranges, as a tool to estimate their conservation status. The objective of this study was to review known species distribution and propose potential and conservation status for *Salvia* species in Michoacán sState using Ecological Niche Models (ENM). We reviewed taxonomic studies for *Salvia* in Michoacán to compile an initial species checklist, built upon with recently-described species; all the specimens deposited in the National Herbarium were reviewed. The collection data allowed us to build niche models of *Salvia* species reported for Michoacán. ENM were generated for the species listed using Maxent. In order to minimise collinearity, environmental variables were selected using a Pearson correlation test. Individual models were statistically evaluated and the potential distribution models for each individual species were stacked to obtain the map of richness potential distribution in the State. A total of 66 species of *Salvia* are listed for Michoacán; however, ENM could only be constructed for 42 of those with ≥ 5 specimens. The environmental variable that most strongly contributed to the models was annual average temperature. The models estimated that *Salvia* species occupy an area of 23,541 km^2^ in the State, 72% in the Trans-Mexican Volcanic Belt and a second richest ecoregion is the Sierra Madre del Sur. Although only 3% of the potential distribution area for *Salvia* in Michoacán is within Protected Areas (PAs), nonetheless, no PA includes rare species. It will therefore be necessary to consider new protection areas or expand existing ones in order to adequately conserve *Salvia* richness and rarity in the State.

## Introduction

Numerous studies document species richness in several regions all over the world; unfortunately, knowledge on the geographic ranges for most of these species is lacking. The few existing reports are often biased by collecting at easily-accessible regions, whereas remote areas are under-sampled. Incomplete sampling has a direct effect on spatial distribution conceptions and negatively influence biogeographic interpretations (Ponder et al. 2001, Whittaker et al. 2005, Boria et al. 2014).

Although much effort has been made to assess species geographic distribution in Mexico, nonetheless, documenting geographical ranges occupied by plants species has been sparse (Villaseñor and Téllez-Valdés 2004). This is unfortunate, given Mexico´s high endemism degree (Villaseñor 2016) and the many useful plants (Caballero and Cortés 2001). One such group is *Salvia* (Lamiaceae), with many useful species, but distribution is not well known.

*Salvia* is the largest genus of the Lamiaceae; worldwide, it is represented by about 1000 species (Walker et al. 2004, Martínez-Gordillo et al. 2017), 306 of which are distributed in Mexico with ca. 77.1% of endemism in the country (Ramamoorthy and Elliot 1998, Martínez-Gordillo et al. 2017). In addition to their species richness and endemism, several species are of economic, medicinal or ornamental importance (Cahill 2003, Moss et al. 2010, Munguía-Lino et al. 2010, Martínez-Gordillo et al. 2013, Akaberi et al. 2016, Martínez-Gordillo et al. 2017). In Mexico, the most emblematic species in traditional medicine include *Salvia divinorum* Epling & Játiva for its psychoactive activity, *Salvia mexicana* L. and *Salvia tiliifolia* Vahl as anti-inflammatory and *Salvia hispanica* L. as an alimentary supplement (Cahill 2003, Argumedo et al. 2003, Maqueda et al. 2015, González-Chávez et al. 2018). Despite their importance in biodiversity and practical uses, the vast majority of the species' environmental factors that determine their distribution are not known (Martínez-Gordillo et al. 2017).

The genus' taxonomy has been adequately studied for the western Mexican State of Michoacán. Cornejo-Tenorio and Ibarra-Manríquez (2011) reported a total of 64 native species. Later, Lara-Cabrera et al. (2016) in a Lamiaceae-wide list, report only 62 species, through distinct species synonymy conceptions and additions not previously mentioned or recently-described new species. Finally, in a perusal of the Lamiaceae family for Mexico, Martínez-Gordillo et al. (2017) reported 306 species of *Salvia* for the country, 69 distributed in Michoacán. Thus, *Salvia*'s species richness in Michoacán ranges between 62 and 69 species. Several taxonomic studies (Table 1) of infra-generic groups within *Salvia* distributed in the State have also reviewed Michoacán species; S. sect. Sigmoideae (Espejo-Serna and Ramamoorthy 1993), S. sect. Membranaceae (González-Gallegos 2014) and S. sect. Scorodoniae (Olvera-Mendoza et al. 2017). Of equal interest is the taxonomic treatment by González-Gallegos et al. (2016) for the Lamiaceae in the State of Jalisco and species distributed throughout adjacent Michoacán.

The State of Michoacán is amongst the top five most *Salvia*-rich states in Mexico (Cornejo-Tenorio and Ibarra-Manríquez 2011). According to Martínez-Gordillo et al. (2017), six species are endemic to the State (*Salvia cyanantha* Epling, *Salvia gravida* Epling, *Salvia indigocephala* Ramamoorthy, *Salvia madrigalii* Zamudio & Bedolla, *Salvia plurispicata* Epling and *S. synodonta* Epling). *Salvia* species are abundantly distributed in the north-eastern region of the State, mainly in temperate and warm regions, at altitudes ranging from 1500 to 3000 m above sea level (Cornejo-Tenorio and Ibarra-Manríquez 2011, Lara-Cabrera et al. 2016). In addition to its great diversity, several *Salvia* species occurring in Michoacán are being investigated for their economic potential in essential oil production, medicinal and ornamental potential (Cornejo-Tenorio and Ibarra-Manríquez 2011, Martínez-Gordillo et al. 2013).

Despite extensive research on the taxonomy of *Salvia* in Michoacán, the rheographic ranges that these species occupy and their relationship with environmental variables has been little explored. Delimiting suitable geographic ranges for species is fundamental, directly underpinning a range of biodiversity and ecosystem function indicators (Jetz et al. 2019), aiding too to traditional morphology-based taxonomy, faced with great challenges in complex taxa (Li et al. 2019). Ecological niche models (ENM) are one of the most commonly used methods for estimating biodiversity patterns, enabling the estimation of species' distributions by association of environmental predictors and presence data (Peterson et al. 2011). In addition, ENM have been widely used in studies dealing with macroecology, conservation, niche evolution, climate change and potential for expansion of invasive species, to mention a few (Hijmans and Graham 2006, Warren et al. 2008, Peterson et al. 2011).

The present research aims to better characterise the distribution of *Salvia* in Michoacán by: 1) updating the known distribution of the genus in the State, 2) using ENM to determine the potential geographic distribution and the environmental variables influencing habitat suitability for these species, 3) comparing the known and potential distribution of the genus to pinpoint areas for further botanic collection and test performance of the ENM and 4) assessing the conservation status both at genus and species level, by analysing habitat loss and coverage of *Salvia* distribution in state-protected natural areas. This approach will identify regions of importance for the conservation of this important genus of the Mexican flora.

## Material and methods

### Species checklist establishment

We reviewed the most relevant *Salvia* taxonomic papers from Michoacán [Espejo-Serna and Ramamoorthy (1993), Bedolla-García et al. (2011), Cornejo-Tenorio and Ibarra-Manríquez (2011), Iltis et al. (2012), Martínez-Gordillo et al. (2013), González-Gallegos (2014), González-Gallegos et al. (2016), Lara-Cabrera et al. (2016), Martínez-Gordillo et al. (2017) and Zamudio and Bedolla-García (2018)] and obtained a summarised species list for this study.

Given the taxonomic complexities of the group, with frequent synonymy changes, we only considered the species that were reported in at least two of those studies and that could be corroborated with herbarium specimens. Discrepancies in the *Salvia* list include the number of species, 78 in three papers (Cornejo-Tenorio and Ibarra-Manríquez 2011, Lara-Cabrera et al. 2016, Martínez-Gordillo et al. 2017), although this number is slightly overestimated; for example, *Salvia arbuscula* Fernald is recognised by Cornejo-Tenorio and Ibarra-Manríquez (2011), but considered as a synonym to *S. iodantha* Fernald by Lara-Cabrera et al. (2016). Martínez-Gordillo et al. (2017) do not even include the species for the State. Finally, we updated the list including a recently-described species *S. madrigalii* Zamudio and Bedolla-García (2018).

### Occurrence data

*Salvia* occurrences information for Michoacán was obtained from databases of the National Biodiversity Information System of Mexico (SNIB-REMIB) of the Comisión Nacional para el Conocimiento y Uso de la Biodiversidad (CONABIO), the digital repository of the National Herbarium of Mexico (MEXU-UNIBIO) of the Instituto de Biología, Universidad Nacional Autónoma de México (UNAM) and through the review of taxonomic studies. All specimens deposited in the National Herbarium (MEXU) were reviewed, verifying species identification (see Suppl. material 1).

### Cleaning of database

The database was corrected following the recommendations by Chapman (2005) and Castillo et al. (2014) with the following procedure: 1) correct identification of the specimens as verified by specialists, 2) eliminate synonym and duplicates, 3) geo-referencing the localities lacking this information in the specimen label, in Google Earth (https://www.google.com/earth/), using locality name and description and finally 4) eliminate correlated records through pattern analysis.

Pattern analysis was applied to the data for all species using the Ilwis v.3.4 programme (http://52north.org/ilwis). This analysis allowed us to estimate the distance at which the collecting points are not correlated (Cruz-Cárdenas et al. 2014a). The distance obtained from the pattern analysis was used to filter the uncorrelated data through the R spThin package (Aiello-Lammens et al. 2015, R Development Core Team 2016), integrated in the Data module Process Occurrence in Wallace v.1.0.6 (Kass et al. 2018). This analysis reduced sampling *bias* by eliminating records per geographic distance, which significantly influences the models performance (Boria et al. 2014).

In addition to the review of the specimens in the herbarium, specimens cited in taxonomic works of the study group were also considered. For the species and specimens with taxonomic circumscription issues, the specimens were individually evaluated to determine whether or not to include them in the analyses. The outliers identified in the pattern analysis were reviewed to confirm the identity from a genus specialist; the specimens that were not approved by the specialist, were excluded from the database.

### Known distribution

The polygon for the state of Michoacán was divided into 118 grid-squares of 15' latitude and 15' longitude; a finer scale grid would have resulted in no species being represented. Thus 15' x 15' grid was a compromise between the distribution, collection effort and the number of grids. Geographic information was analysed in ArcGIS 10.2 (ESRI 2013) to produce a known distribution map and intersecting the grids with the number of species recorded in each grid. Grids were subsequently grouped per species number.

### Environmental data

Fifty eight environmental variables were considered, with a resolution of 30 arc seconds, a pixel size of about 1 km^2^ (Table 2); 26 of them were climatic (Hijmans et al. 2005), nine topographic, nine edaphic and 14 included remote sensing data (Cruz-Cárdenas et al. 2014b). However, it is known that the use of a large number of variables influences the final results of the models, making it necessary to select only those that are most significant (Peterson and Nakazawa 2008, Varela et al. 2014). To reduce the total number of variables included in models and to minimise multicollinearity amongst the 58 variables, their correlation values (Pearson) were evaluated using NicheToolsBox (Osorio-Olvera et al. 2018); uncorrelated variables were included in the analysis and correlated variables (*r > 0.85*) were excluded from the analysis.

### Ecological niche models

Species distribution result from several factors; amongst the most important ones are environmental variables (A), biotic component (B) and the set of sites that has been accessible to the species (M). Soberón and Peterson (2005) illustrated the interaction of these factors in a diagram called BAM. We used the WWF Ecoregions following Olson et al. (2001) to delimit the accessible area for all species (M in the BAM diagram, Soberón and Peterson 2005), considering only the ecoregions with occurrence records of *Salvia* in Michoacán. Selected ecoregions were then cut, using the State's polygon as a limit.

Evaluating models using spatially independent data improves model configurations and balances their complexity. There are several methods for identifying optimal model configuration (Muscarella et al. 2014, Hijmans et al. 2017). Here, we used the package ENMeval v.0.1.2 in R (Muscarella et al. 2014) to determine the best performance models, run with multiple regularisation (MR) values, from 1 to 4 (in a 1 increment), with four different feature class combinations (L, LQ, H, LQH, where L = Linear, Q = Quadratic, H = Hinge). ENMeval provides multiple evaluations that allow the identification of the optimal model configuration (Muscarella et al. 2014).

Maxent 3.4.1 software (Phillips et al. 2017) was used to run the ENM. Maxent has gained popularity for ENM of many plant and animal species due to its simplicity in configuration. Models were constructed by changing the regularisation values and the feature class, following the results from ENMeval analysis. The default options of clamping and extrapolate were omitted, in order to avoid extrapolation of the extreme values of variables (Elith et al. 2011). Software was set to use 75% of the data to run the analysis and 25% to validate the model (Phillips et al. 2006), only for species with more than five records, the minimum number required in Maxent (Hernandez et al. 2006). For a few records species (5-20), we decided to construct the models using the basic configuration of Maxen (Phillips et al. 2017) and not the feature classes and regularisation multiplier, since these generally result in low Area under the Curve (AUC) values and overprediction. Species with less than five records were considered for generating the map of known richness and are considered amongst the rare species.

Results from Maxent were then processed in ArcGIS 10.2 (ESRI 2013) to obtain binary maps, using the maximum training sensitivity plus specificity as the cutoff threshold (Liu et al. 2016). These binary maps were added with the Algebra map tool to obtain a richness map of *Salvia* species. In addition, the known richness in the State was obtained from collection records in digitised databases, compared with the potential richness obtained through the models. Mexdem (digital elevation model) altitude layer in ArcGIS was used to extract the altitude values of each record of *Salvia* species.

### Training/testing partitioning and model evaluation

We partitioned the data into training and testing groups (k-fold cross validation), allowing us to evaluate models’ performance (Peterson et al. 2011). We chose to use "block (k-4)", one of the options implemented in ENMeval (Muscarella et al. 2014), which divides the presence data according to its longitude and latitude. This method is one of the most recommended, since it provides better spatial independence of the data (Radosavljevic and Anderson 2014, Fourcade et al. 2018).

The models were evaluated using the AUC, a value that is part of Maxent's results. AUC values range from 0 to 1, where values close to 1 indicate models with perfect discrimination ability and values less than 0.5 indicate that the model is no better than a randomly-generated model (Peterson et al. 2011, Fourcade et al. 2018). Individual models were also evaluated by the binomial test analysis and partial-ROC (receiver operating characteristic), considering a significance value *< 0.05*. This allowed the evaluation of the models’ ability to predict the largest possible number of independent points amongst those that were not used to create the model. The tests were carried out using the R statistical package and NicheToolBox (Osorio-Olvera et al. 2018).

### Conservation status

To assess the conservation status of the genus *Salvia*, habitat loss and the area currently within a Protected Area (PA) were analysed along the potential distribution of the genus in the State of Michoacán. To analyse the habitat loss of the genus, a land use map of Series I and VI of the National Institute of Geography and Informatics (INEGI 1997, INEGI 2017) was used. Maps were then reclassified into two categories: vegetated and non-vegetated (Staude et al. 2019); (1) Specifically, those areas that represent some forest cover within the State polygon were selected (e.g. oak forest, coniferous forest, evergreen forest, deciduous forest, sub-deciduous forest and xerophytic scrub). (2) To estimate the degree of protection of *Salvia*, the map of potential distribution was overlapped with the map of protected natural areas of Mexico (CONANP 2010).

## Results

### Species and occurrence data

A list of 66 species was obtained for the Mexican State of Michoacán (Suppl. material 2) from bibliographic review of *Salvia*. Fifty two species are reported as endemic to Mexico and three species are restricted to the State (*S. madrigalii*, *S. subobscura* and *S. synodonta*). A complete dataset record of 3,093 was later reduced to 1,925 records after data cleaning. Of these records, 1,836 correspond to the 42 *Salvia* species with pattern analysis and, finally, 404 records were used to build the ENMs.

### Patterns of distribution of *Salvia* in Michoacán

#### Known distribution

**Figure 1.**
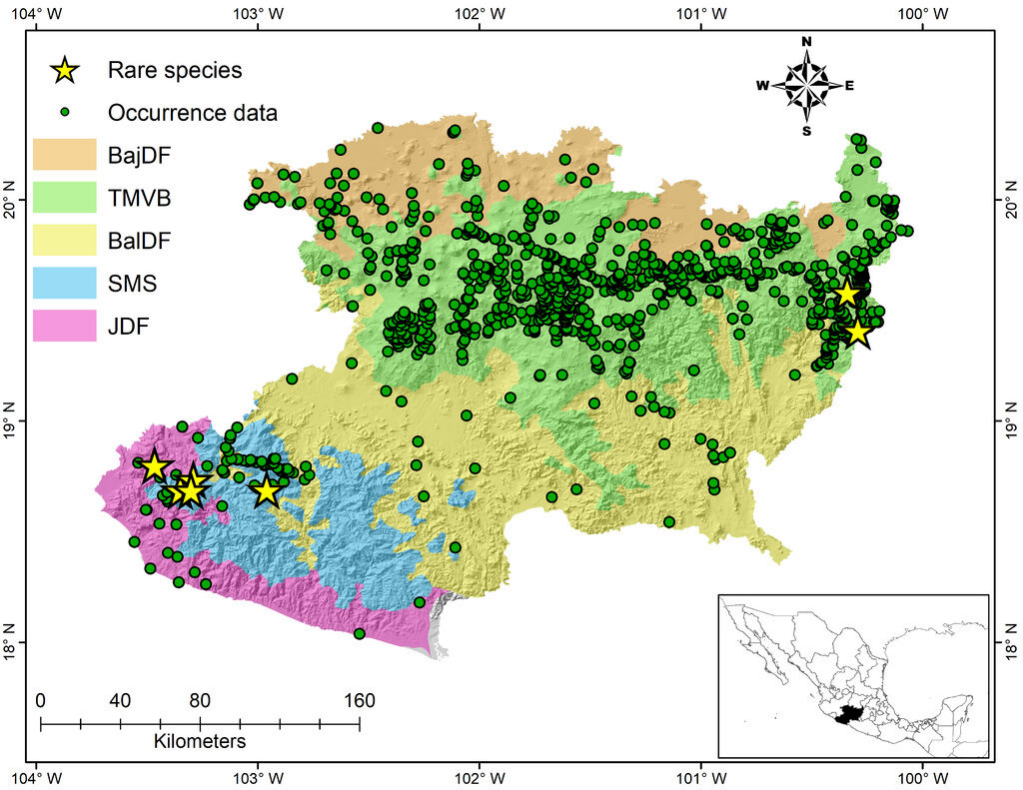
Known distribution of the genus *Salvia* based on collecting effort (green points) in Michoacán. The yellow stars indicate the location of species that have only one record in Michoacán. Colours show the boundaries of each ecoregion. BajDF: Bajío Dry Forests, TMVB: Trans-Mexican Volcanic Belt, BalDF: Balsas Dry Forests, SMS: Sierra Madre del Sur, JDF: Jalisco Dry Forest

The political territory of Michoacán comprise five ecoregions; *Salvia* has been reported in all (Fig. 1). Of these the Trans-Mexican Volcanic Belt (TMVB) contained the largest number of species (56 species), followed by 21 species in the Sierra Madre del Sur (SMS) and 14 in the Jalisco Dry Forest (JDF).

The *Salvia* endemic species to the state occupy a very restricted area, as exemplified by *S. madrigalii*, having being reported at only three sites in the Morelia Municipality, in north-eastern Michoacán. A similar case is *S. subobscura* in the south, known from only two locations in the Chinicuila Municipality. Eight in the restricted species group are here regarded as rare species (< 3 records), known only from one locality (the type locality, Fig. 1), five from western Michoacán, in Chinicuila Municipality (*Salvia acerifolia* B.L. Turner, *Salvia decora* Epling, *Salvia fusca* Epling and *Salvia subhastata* Epling) and Coalcomán (*Salvia cyanantha* Epling) and two (*Salvia atropaenulata* Epling and *Salvia filifolia* Ramamoorthy) from the northeast, in Zitácuaro and Ocampo Municipalities.

#### Potential distribution

Individual models for *Salvia* species indicate that 59% (34,784.6 km^2^) of the State’s total area (58,836.95 km^2^) is environmentally suitable to harbour its species. The species with the largest and smallest distribution areas were *Salvia clinopodioides* Kunth with 19,895.3 km^2^ and *S. madrigalii* with only 16.8 km^2^ (Table 3).

**Figure 2.**
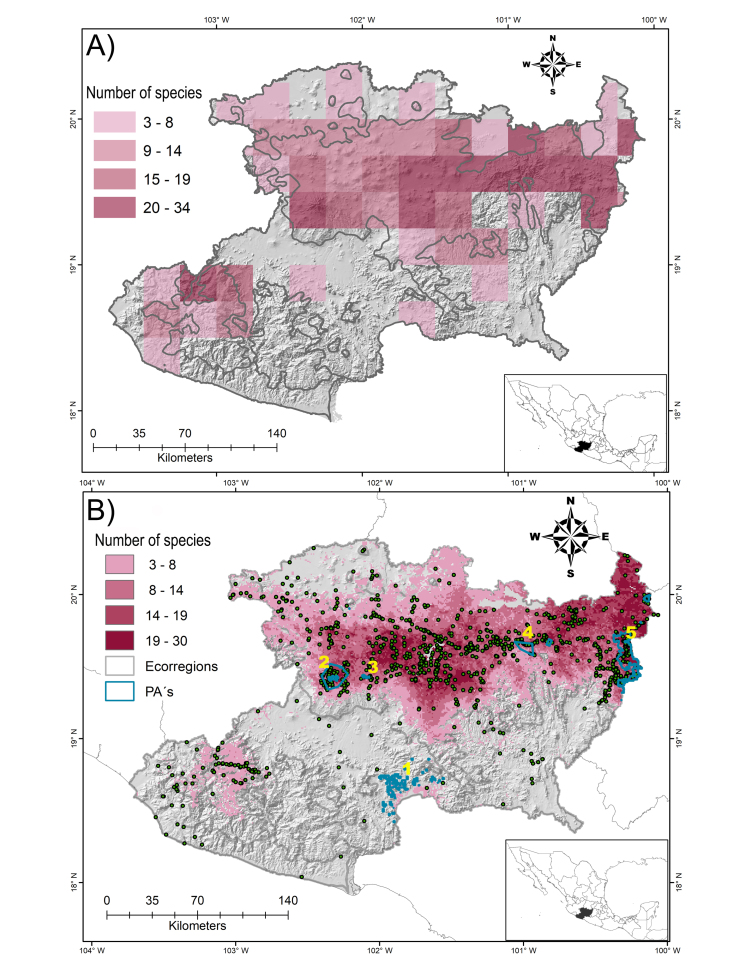
Known and potential distribution of the genus *Salvia*. **A.** The State of Michoacán, Mexico, divided into 15′ × 15′ squares showing the known distribution of the genus *Salvia* in the State; **B.** Potential distribution of the genus *Salvia* in Michoacán (pink colour). Darker colours correspond to areas with more assemblage of *Salvia* species. The points indicate the collection sites. Blue lines show the boundaries of the Protected Areas and grey boundaries correspond to the limits of the ecoregions. 1: Zicuirán-Infiernillo, 2: Pico de Tancítaro, 3: Barranca del Cupatitzio, 4: Insurgente José María Morelos, 5: Mariposa Monarca

The known richness in Michoacán, depicted in 15' x 15' grids (Fig. 2A), matches the potential richness (the stacking of 42 ENMs, Fig. 2B). The cutoff point for the richness map was determined by the lowest percentage of omission errors (0.7%), obtained using all 1,925 records of the 66 *Salvia* species here considered. Below this threshold, the suitable conditions for *Salvia* species cover an area of 23,541 km^2^ (Fig. 2), 72% in the Trans-Mexican Volcanic Belt portion of the state.

**Figure 3.**
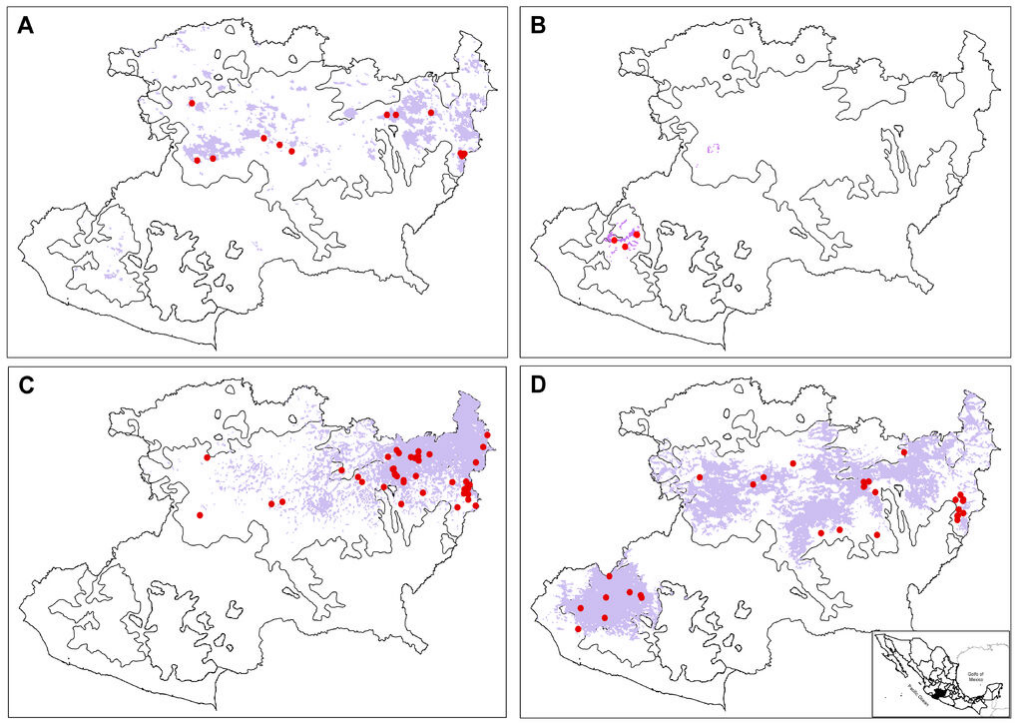
Examples of potential distribution areas of species of *Salvia* endemic to Mexico (lilac colour) and representative of the Michoacán ecoregions (black boundaries within the State of Michoacán). **A.** *Salvia albocaerulea*; **B.** *Salvia protracta*; **C.** *Salvia plurispicata* (endemic to Michoacán); **D.** *Salvia dichlamys*. The red circles show the points of occurrence of the species.

Niche models are here reported for four *Salvia* species endemic to Mexico inhabiting Michoacán (Fig. 3); *Salvia albocaerulea* Linden (Fig. 3A) and *Salvia protracta* Benth (Fig. 3B) restricted to the TMVB and the SMS, respectively. *Salvia plurispicata* (Fig. 3C) and *Salvia dichlamys* Epling with wide distribution thoughout the State, occurring in the five ecoregions (Fig. 3D).

The altitude for these species is variable, although 81% of them are distributed between 2000 and 2500 m a.s.l. The number of species decreases, both at lower (< 500 m) and higher altitudes (> 3000 m).

### Ecological niche models

A total of 26 uncorrelated variables (of the initial 58) were considered to run the ENMs for each *Salvia* species with sufficient (> 5) records, including eight climatic variables, seven topographic, eight edaphic and three from remote sensing data (Table 2). The variable of highest contribution value (42% of the models) was the Annual mean temperature (bio01) based on the Jackknife test provided by Maxent, with contribution percentages between 36 and 77.5%. The second most important variable was the Magnesium content (mexmg), important in 14% of the *Salvia* models, with contribution percentages from 19 to 69% and the least informative variable was the Precipitation of the coldest quarter (bio19) with 0.5%.

We obtained 42 ENMs of *Salvia* species in Michoacán (Suppl. material 3) out of 66 species, based on 404 spatially-uncorrelated records (Table 3); 10 of these models are considered exploratory having less than the five records required to be validated statistically. In addition, only three models were run using the regularisation values and the feature class obtained from the evaluation with ENMeval (Suppl. material 4); all the other models were obtained using the default parameters as specified in Maxent.

All models (made by default setting and configured) showed good performance, AUC values above 0.84 were considered good, 19% (AUC > 0.8) and 81% excellent (AUC > 0.9, Table 3). The validation of the models with the binomial test indicated that 42% models had statistically-significant prediction capacity (0.53-0.93, *p-value* = 0.03-3.46 x 10^-14^ respectively), while 58% of the models were not statistically significant. In contrast, the Partial-ROC tests were statistically significant (*p* < 0.05) for all species, with values between 1.13 and 1.99 (mean 1.58, Table 3).

### Conservation status

The geographic area obtained with the assembly of models for the *Salvia* species has been profoundly affected by land use change. It has been estimated that 22% of the area has been lost in the last 20 years (1997 - 2017); TMVB ecoregion is the most severely affected, with a reduction of about 43% loss of its primary vegetation. The least affected primary vegetation loss ecoregions are JDF and the SMS (less than 10%).

Ten Protected areas in Michoacán harbour potential distribution areas of *Salvia* species. Overall, these PAs include 3.3% of the potential distribution predicted. Eight of these PAs are located in the TMVB ecoregion; unfortunately, the SMS ecoregion does not register any PA. A total of 55% *Salvia* species in the State are represented in the PAs; of these, 25 species are distributed in the Monarch Butterfly Reserve (the Monarch Butterfly Hibernation Protection Zone). Only 6% of the rare species include populations within the State's PAs.

## Discussion

### Species and occurrence data

The *Salvia*’s database of Michoacán State includes more than 3,000 records, reduced to 1,925 unique records after eliminating duplicates. Although the database includes a significant number of records for the State, only 39.6% are cited in the referred floristic-taxonomic studies of relevance to the State. The two projects, focused on *Salvia* in Michoacán (Cornejo-Tenorio and Ibarra-Manríquez 2011, Lara-Cabrera et al. 2016), combine up to 701 specimens representing 36.4% of the total database; 685 specimens were collected before 2010 and are currently already included in online databases. Both studies cited a total of 217 reviewed specimens, representing 44% and 31.2%, respectively of those here reviewed. Although they do not explicitly indicate whether the number of specimens cited represent a total sample or a sample of the reviewed specimens, only 39.6% of the total collections for *Salvia* are cited.

Undoubtedly, 1,925 records representing 66 of the 69 species occurring in the State (Martínez-Gordillo et al. 2017), are a good sample of the State's generic diversity, with broad habitat diversity where the genus thrives. Many additional records here documented were reviewed by the group's specialists, although they were not cited in their floristic-taxonomic works. The 1,925 unique records included here are a summary of more than 3,000 specimens kept in about 20 different herbaria, both in Mexico and abroad (Table 1). The National Herbarium (MEXU) at the Instituto de Biología, Universidad Nacional Autónoma de México stands out, housing 64.5% of the total records (1,251); 1,159 of the 1,836 records used for the elaboration and validation of ENMs were at MEXU, constituting the main supply of information and headquarters of the study carried out here.

### Patterns of distribution of *Salvia* in Michoacán

Our results indicate that *Salvia* species in Michoacán preferentially occur in temperate or seasonally-dry forests, the predominating biomes in the State (Gopar-Merino and Velázquez 2016); that is why more than 50% of the surface of the State is suitable for the species. Our results agree with those reported by Lara-Cabrera et al. (2016), which document higher species richness in the TMVB, where coniferous, oak and humid mountain forests abound. Similarly, Cornejo-Tenorio and Ibarra-Manríquez (2011) report that 40 *Salvia* species are distributed in the pine-oak and oak forests. Thus, our ENM are consistent with the previously reported environmental preferences for the genus.

The majority of *Salvia* species act as generalists to environmental conditions throughout their distribution in Michoacán. Only temperature had the highest variable contribution amongst all biomes; temperature is frequently documented as the dominant abiotic driver in determining plant distributions (Körner and Hiltbrunner 2018, Scherrer and Guisan 2019). Mean temperature is associated with plant growth, acting mainly in a gradual manner (Körner et al. 2016), determining whether a plant species will be present (Guisan et al. 1998), particularly in the mountainous landscape, such as is characteristic in the TMVB (Cornejo-Tenorio and Ibarra-Manríquez 2011, Lara-Cabrera et al. 2016). Oddly, precipitation was not a significant variable; a correlation between precipitation and species richness at regional scale in these environments has been well documented, the greater the precipitation, the greater the species richness (Pau et al. 2012). However, precipitation's contribution here is low, probably due to scarce records from the Balsas Dry Forests and JDF (Fig. 2), where rainfall is below 1,000 mm.

Rare species (< 3 records), although not modelled, show a preference for similar environmental conditions as compared to modelled species or widely-distributed species (> 3 records). For example, the mean temperature of rare species (18°C) is slightly higher than that of widely-distributed species (15.7°C), while in precipitation, the mean values are very similar, 1,057 mm for rare species and 1,058 mm for widely-distributed species. Furthermore, several state endemic *Salvia* species require very specific conditions; *S. madrigalii*, *S. synodonta* and *S. cyanantha*, are known only from two sites with practically the same climatic characteristics. The specificity of their habitat, as a result of their restricted distribution, makes these species particularly susceptible to extinction (Isik 2011). Despite having information on the optimal environmental conditions of this species group, very little is known about the biological or physiological aspects that regulate it.

Environmental suitability is indicated for the genus along the SMS (Fig. 2); however, further fieldwork for the region could add important floristic information (Cornejo-Tenorio and Ibarra-Manríquez 2011, Lara-Cabrera et al. 2016), as far as the optimal environmental conditions where they thrive. The above is a particular way for endemics or rare species that would allow us to confirm or improve the distribution patterns reported in this study.

Cornejo-Tenorio and Ibarra-Manríquez (2011) mention altitude as an important variable in the distribution from *Salvia*. The inclusion of this variable in the potential geographic range of *Salvia* in Michoacán confirms its importance in explaining the species distribution. The genus inhabits altitudes from 200 to 3,600 m, as reported by Lara-Cabrera et al. (2016); however, species richness is not evently distributed throughout this altitudinal range, with middle altitudes (2,000-2,500 m) as the richest in species (52 species) and decreasing richness both at lower and higher altitudes. These data coincide with those published by Cornejo-Tenorio and Ibarra-Manríquez (2011), who mention that the highest richness was found above 2,000 m altitude (35 species).

Although the large amount of information in the databases would seem sufficient for ENMs and thus inferring many species distribution, this was not always the case. When performing statistical analyses to develop more robust and precise models, such as eliminating spatial autocorrelation of points to reduce sampling *bias* or to optimise the specific configuration for each model adjusted for each species data (Boria et al. 2014, Radosavljevic and Anderson 2014, Warren et al. 2014), it became clear that we did not have enough independent records. In the case of *Salvia* in Michoacán, despite several works specifically focused on these species (Cornejo-Tenorio and Ibarra-Manríquez 2011, Lara-Cabrera et al. 2016, Zamudio and Bedolla-García 2018), we still did not have sufficient data for most species to develop ENMs under specific configurations. We found that 78% of the purged data for *Salvia* are spatially correlated, which is likely the result of sampling bias to areas of easy access or of particular interest, resulting in several records from a single site for the same species. Due to this strong point correlation, we were able to obtain only three configured models (models whose rigour was increased by adjusting their configuration or balancing their complexity). We corroborate the guidelines by other authors, at least 20 records are necessary to obtain and reach the predictive capacity for models (Stockwell and Peterson 2002, Mateo et al. 2010, van Proosdij et al. 2016). In our case, using few records to obtain regularisation values, we obtained models with low predictive value, resulting in high rates of model overprediction and AUC values below 0.5. For this reason, we too recommended that the tests to obtain regularisation values and make configured models be used only when more than 20 records are available; the larger the number of records, the better the model performance (Stockwell and Peterson 2002) as shown in *Salvia*.

The AUC has been criticised as a tool to evaluate only the performance of presence models, because of its dependence on prevalence; therefore, it is not considered a precise performance index (Lobo et al. 2008, van Proosdij et al. 2016). For this reason, other performance evaluation metrics, such as Partial ROC for continuous data (Peterson and Nakazawa 2008) and the binomial test for binary models (Anderson et al. 2002), were implemented. We included these test results as additional evaluation measures (Table 3). The number of non-significant binomial tests (58% of the models) correspond to the number of species with less than five records. Blair et al. (2013) obtained similar results when using ten records; below this sample size, their binomial tests were not significant (*p* > 0.05). We recommend reaching a minimum number of 20 independent points of occurrence to construct models with better predictive power that pass the test of sensitivity to small samples.

High deforestation rates in the north-central region of Michoacán, is the main cause of habitat loss. The most vulnerable vegetation types are temperate forests, constantly being replaced by avocado orchards (Mas et al. 2017) amongst other threats, thus decreasing the original distribution of many species, as we found here for *Salvia*.

## Implications for conservation

Protected areas represent a key strategy for biodiversity preservation (Hannah et al. 2007, Belote and Wilson 2020); however, most are limited to small geographic areas that do not represent the region's diversity for *Salvia*. Only 3% of its potential distribution is present in PAs, comprising 55% of its species richness. In order to increase the representation of these species within the PAs, it has been proposed to raise the conservation status to larger ecosystems; this approach would allow us to increase the representation of vegetation types and with it, the number of conserved species (Belote and Wilson 2020).

The results point to the TMVB, the SMS and the JDF as priority ecoregions for *Salvia* conservation in Michoacán, where the most species richness and rare species (the only known locality for some of the species) are located. Despite the high species richness, few PAs in the State have been thoroughly sampled (Cornejo-Tenorio and Ibarra-Manríquez 2011). The State PAs (Fig. 2) correspond with only 3% of *Salvia*’s potential distribution area and none of them includes sites for rare species. For example, in the south-western part of the State, no PA is recorded, although nine rare species are recorded in this region and three of them are only known from one locality. Such is the case of *S. acerifolia* (JDF) or *S. cyanantha* and *S. subhastata* (both from the SMS); conservation strategies should be focus on these sites. In the case of the TMVB, seven rare species are recorded and only eight PAs have been decreed; thus, future conservation efforts should aim at their expansion, to include a greater number of species and thereby conserve rare species. This ecoregion is suffering the greatest natural habitats loss (56%). The areas of highest richness identified here (darker areas of Fig. 2) could be used to propose specific sites for the expansion of protected areas.

## Supplementary Material

EDF21191-F8E4-598E-AE2A-F7835E6F59F7 10.3897/BDJ.8.e56827.suppl1 Supplementary material 1 List of specimens of *Salvia* species occurring in the State of Michoacán, Mexico considered in this study. Data type Specimens Brief description Each record is made up of the collector’s last name and collecting number; in parentheses the herbaria where the specimen is stored, followed by the coordinates in decimal degrees. In brackets, the publications in which the records are cited is indicated. 1: Espejo-Serna and Ramamoorthy (1993); 2: Cornejo-Tenorio and Ibarra Manríquez (2011); 3: Iltis et al. (2012); 4: González-Gallegos et al. (2014); 5: González-Gallegos et al. (2016); 6: Lara-Cabrera et al. (2016); 7: recorded in SNIB-REMIB and/or MEXU-UNIBIO databases. File: oo_464475.docx https://binary.pensoft.net/file/464475 Mayra Flores-Tolentino, José Luis Villaseñor and Sabina Lara-Cabrera

470B08D6-0976-56D5-92F0-C3321CCD1D0B 10.3897/BDJ.8.e56827.suppl2 Supplementary material 2 Species of *Salvia* recorded in the State of Michoacán and considered in this study Data type List of species Brief description The number of records obtained for each species after cleaning the data from the SNIB-REMIB and MEXU-UNIBIO databases are indicated. File: oo_464477.pdf https://binary.pensoft.net/file/464477 Mayra Flores-Tolentino, José Luis Villaseñor and Sabina Lara-Cabrera

FDB7172A-4796-54F6-841C-ABC694E1317C 10.3897/BDJ.8.e56827.suppl3 Supplementary material 3 Ecological niche models of 42 *Salvia* species. Data type Images Brief description Ecological niche models of 42 *Salvia* species. The points on each map show the collecting localities. File: oo_464478.pdf https://binary.pensoft.net/file/464478 Mayra Flores-Tolentino and José Luis Villaseñor

2BFCEC61-E756-58A5-8936-2EF5807E91A3 10.3897/BDJ.8.e56827.suppl4 Supplementary material 4 Configuration of Maxent Data type Evaluation metrics Brief description Evaluation metrics of Maxent ENMs generated with the ENMeval programme for the three species of *Salvia* for which the statistical validation could be carried out. FC= Feature class (Logistic); MR= Multiple regularisation; ΔAICc= Delta Akaike Information Criterion. File: oo_433111.doc https://binary.pensoft.net/file/433111 Mayra Flores-Tolentino and José Luis Villaseñor

## Figures and Tables

**Table 1.** Floristic and taxonomic studies accounting for species diversity of *Salvia* in the Mexican State of Michoacán.

**Study**	**Species in** **Michoacán**	**Total number of records cited for Michoacán**	**Number of records** **in *Salvia*’s data base**
Espejo-Serna and Ramamoorthy (1993)	4	52	52
Cornejo-Tenorio and Ibarra-Manríquez (2011)	64	493	439
González-Gallegos (2014)	3	5	1
González-Gallegos et al. (2016)	14	189	97
Lara-Cabrera et al. (2016)	62	695	479
Martínez-Gordillo et al. (2017)	69	None	None
Olvera-Mendoza et al. (2017)	3	19	6
*Salvia*’s data base (this study)	70	768	1,925

**Table 2.** Variables used to estimate the ecological niche models of *Salvia* species in Michoacán. The variables with the highest and lowest contribution in the ENMS of *Salvia* species are highlighted in bold.

**Type**	**Variable**
*Climatic*	**bio01 (Annual mean temperature)**
	bio02 (Average daytime variation)
	bio03 (Isothermality)
	bio14 (Precipitation of driest month)
	bio15 (Seasonality of precipitation)
	bio18 (Precipitation of the warmest quarter)
	bio19 (Precipitation of the coldest quarter)
	evaanual (Annual real evapotranspiration)
*Topographic*	aspect (Orientation 0° to 90°)
	convrgin (Convergence index)
	dah (Diurnal anisotropic heating)
	mexslope (Slope)
	runoff (Flow)
	twi (Topographic moisture index)
	vrm (Vector rugosity measure)
*Edaphic*	mexca (Calcium)
	mexce (Electrical conductivity)
	mexco (Organic carbon)
	mexk (Potassium)
	**mexmg (Magnesium)**
	mexmo (Organic material)
	mexna (Sodium)
	mexras (Sodium absorption radius)
*MODIS	modismar (Normalised index of vegetation March)
	modissep (Normalised index of vegetation September)
	hummodis2009 (Normalised index of vegetation humid months)

**Table 3.** Species of *Salvia* in Michoacán for which ecological niche models could be constructed. The number of records used to build the models and the surface estimated by the models are indicated. The values of the AUC and Partial-ROC tests are indicated for each species. Bold type indicates species that did not pass the binomial test (*p* > 0.05). The exploratory models are marked with an asterisk.

Species	Total records/records to build the models	Potential area (km^2^)	AUC	Partial ROC	Binomial test
*Salvia albocaerulea* Linden	14/5	3286.3	0.972	1.7	**0.193 (0.64)**
*Salvia amarissima* Ortega	15/7	8281.8	0.934	1.90	**0.050 (0.49)**
*Salvia assurgens Kunth	44/4	3727.1	0.962	1.38	**0.183 (1)**
*Salvia carnea Kunth	6/4	1576.0	0.986	1.83	**0.355 (0.29)**
*Salvia clinopodioides* Kunth	47/12	19895.3	0.837	1.56	0.913 (1.16 x 10^-10^)
*Salvia curviflora* Benth.	8/5	3388.1	0.971	1.91	**0.473 (0.06)**
*Salvia dichlamys* Epling	32/15	11831.4	0.89	1.29	**0.260 (0.69)**
*Salvia elegans* Vahl	130/25	11912.2	0.874	1.56	0.795 (3.46 x 10^-13^)
*Salvia fulgens* Cav.	95/10	9306.3	0.909	1.67	0.770 (1.36 x 10^-11^)
*Salvia gravida Epling	8/3	1982.4	0.988	1.97	0.607 (0.02)
*Salvia gesneriiflora* Lindl. & Paxton	57/17	3516.7	0.985	1.59	0.575 (0.006)
*Salvia helianthemifolia* Benth.	48/7	2873.0	0.979	1.93	0.930 (4.55 x 10^-13^)
*Salvia hispanica L.	23/4	1853.2	0.974	1.23	**0.003 (1)**
*Salvia iodantha* Fernald	138/20	7989.4	0.896	1.49	0.556 (0.002)
*Salvia laevis* Benth.	57/18	12436.1	0.932	1.59	0.887 (3.73 x 10^-11^)
*Salvia languidula Epling	9/4	291.8	0.995	1.85	**0.009 (0.98)**
*Salvia lasiocephala* Hook. & Arn.	34/14	8340.8	0.934	1.17	**0.075 (1)**
*Salvia lavanduloides* Kunth	102/15	16136.8	0.883	1.25	0.647 (6.33 x 10^-6^)
*Salvia leptostachys* Benth.	16/5	2505.2	0.987	1.46	**0.063 (0.89)**
*Salvia longispicata* M. Martens & Galeotti	76/13	12802.5	0.928	1.41	0.578 (0.003)
*Salvia longistyla* Benth.	37/8	18161.9	0.926	1.45	0.712 (5.19 x 10^-5^)
*Salvia madrigalii Zamudio & Bedolla	4/3	16.8	1	1.99	**0.473 (0.06)**
*Salvia melissodora* Lag.	11/5	2705.5	0.972	1.61	**0.000 (1)**
*Salvia mexicana* L.	206/24	7161.9	0.861	1.51	0.508 (0.03)
*Salvia microphylla* Kunth	38/6	12920.7	0.9	1.31	0.521 (0.03)
*Salvia misella* Kunth	55/19	10142.5	0.854	1.23	**0.258 (0.84)**
*Salvia mocinoi* Benth.	35/14	10954.4	0.925	1.71	0.793 (1.05 x 10^-5^)
*Salvia patens* Cav.	20/12	2008.3	0.981	1.94	0.529 (0.04)
*Salvia plurispicata* Epling	55/13	6165.0	0.963	1.69	0.663 (0.0003)
*Salvia polystachia* Cav.	96/11	18053.7	0.838	1.39	0.768 (2.31 x 10^-11^)
*Salvia protracta Benth.	4/3	171.0	0.997	1.99	**0.0 (1)**
*Salvia prunelloides* Kunth	13/5	707.6	0.993	1.71	**0.129 (0.77)**
*Salvia purpurea* Cav.	62/10	9902.8	0.899	1.32	**0.435 (0.24)**
*Salvia reflexa Hornem.	4/4	5217.0	0.948	1.87	**0.0 (1)**
*Salvia reptans* Jacq.	55/12	7664.7	0.952	1.47	0.577 (0.004)
*Salvia rhyacophila (Fernald) Epling	6/4	2073.6	0.985	1.95	**0.0 (1)**
*Salvia sessei* Benth.	34/6	16430.3	0.877	1.32	**0.282 (0.73)**
*Salvia setulosa Fernald	6/4	912.5	0.99	1.97	**0.0 (1)**
*Salvia stachyoides Kunth	11/4	2404.5	0.976	1.66	**0.200 (0.73)**
*Salvia thyrsiflora* Benth.	70/15	6612.8	0.882	1.13	**0.195 (1)**
*Salvia tiliifolia* Vahl	40/7	12192.5	0.913	1.25	**0.282 (0.73)**
*Salvia uruapana* Fernald	15/8	7186.5	0.933	1.21	**0.076 (0.81)**

## Hosting institution

National Autonomous University of Mexico, Mexico, City.
