# Supplementary material for: Distribution, richness and conservation of the genus Salvia (Lamiaceae) in the State of Michoacán, Mexico
Source: Biodivers Data J. 2020 Oct 29;8:e56827. doi: 10.3897/BDJ.8.e56827 (PMC7644648; doi:10.3897/BDJ.8.e56827)
Supplement: Supplementary material 1 — List of specimens of Salvia species occurring in the State of Michoacán, Mexico considered in this study. [file bdj-08-e56827-s001.docx]

**Supplementary file 2. Species of *Salvia* recorded in the state of Michoacan and considered in this study.**

The number of records obtained for each species after cleaning the data from the SNIB-REMIB and MEXU-UNIBIO databases are indicated. *Species endemic to Mexico and **Species endemic to Michoacán. B: Bosencheve, BC: Barranca de Cupatitzio, CG: Cerro de Garnica, IMM: Insurgente José Maeía Morelos, MM: Mariposa Monarca, PT: Pico de Tancítaro,

| **Species** | **Records** | **PAs** |
| --- | --- | --- |
| **Salvia acerifolia* B.L. Turner | 1 |  |
| **Salvia albocaerulea* Linden | 14 | MM |
| **Salvia amarissima* Ortega | 15 | IJMM, MM |
| **Salvia assurgens* Kunth | 46 | IJMM, MM |
| **Salvia atropaenulata* Epling | 1 | MM |
| **Salvia breviflora* Moc. & Sessé ex Benth. | 3 |  |
| *Salvia carnea* Kunth | 21 | MM |
| **Salvia chalarothyrsa* Fernald | 3 |  |
| **Salvia clinopodioides* Kunth | 47 | CG |
| **Salvia concolor* Lamb. ex Benth. | 3 | MM |
| **Salvia curviflora* Benth. | 8 | MM |
| **Salvia cyanantha* Epling | 2 |  |
| **Salvia decora* Epling | 1 |  |
| **Salvia dichlamys* Epling | 32 |  |
| **Salvia elegans* Vahl | 130 | MM, PT |
| **Salvia filifolia* Ramamoorthy | 1 |  |
| **Salvia fluviatilis* Fernald | 3 |  |
| **Salvia fulgens* Cav. | 95 | B, MM, PT |
| **Salvia fusca* Epling | 1 |  |
| **Salvia gesneriiflora* Lindl. & Paxton | 57 | B, MM, PT |
| **Salvia glechomifolia* Kunth | 2 |  |
| **Salvia gravida* Epling | 8 |  |
| **Salvia helianthemifolia* Benth. | 48 | MM |
| **Salvia hirsuta* Jacq. | 4 | MM |
| *Salvia hispanica* L. | 23 |  |
| **Salvia indigocephala* (Epling) Ramamoorthy | 2 |  |
| **Salvia iodantha* Fernald | 147 | IJMM, MM, PT |
| **Salvia laevis* Benth. | 57 | MM |
| **Salvia languidula* Epling | 9 |  |
| *Salvia lasiocephala* Hook. & Arn. | 34 |  |
| *Salvia lavanduloides* Kunth | 102 | B, MM, PT |
| **Salvia leptostachys* Benth. | 16 | IJMM |
| *Salvia leucantha* Cav. | 4 |  |
| **Salvia longispicata* M. Martens & Galeotti | 76 |  |
| **Salvia longistyla* Benth. | 37 | BC, IJMM |
| ***Salvia madrigalii* Zamudio & Bedolla | 4 |  |
| **Salvia melissodora* Lag. | 11 |  |
| **Salvia mexicana* L. | 206 | B, BC, IJMM, MM, PT |
| *Salvia microphylla* Kunth | 38 | B, MM, PT |
| *Salvia misella* Kunth | 55 | IJMM, MM |
| *Salvia mocinoi* Benth. | 35 | IJMM, MM, PT |
| **Salvia patens* Cav. | 20 | MM |
| **Salvia plurispicata* Epling | 55 | B, CG, MM, PT |
| *Salvia polystachia* Cav. | 96 | MM |
| **Salvia protracta* Benth. | 4 |  |
| **Salvia prunelloides* Kunth | 13 | MM |
| **Salvia pulchella* DC. | 3 |  |
| **Salvia purepecha* Bedolla, S. Lara Cabrera & Zamudio | 3 | IJMM |
| *Salvia purpurea* Cav. | 62 |  |
| **Salvia ramamoorthyana* Espejo | 1 |  |
| *Salvia reflexa* Hornem. | 4 |  |
| *Salvia regla* Cav. | 2 | PT |
| *Salvia reptans* Jacq. | 55 |  |
| **Salvia rhyacophila* (Fernald) Epling | 6 |  |
| **Salvia roscida* Fernald | 2 |  |
| **Salvia sessei* Benth. | 34 |  |
| **Salvia setulosa* Fernald | 6 | CG |
| **Salvia stachyoides* Kunth | 17 | MM |
| **Salvia subhastata* Epling | 1 |  |
| ***Salvia subobscura* Epling | 2 |  |
| **Salvia subpatens* Epling | 3 |  |
| ***Salvia synodonta* Epling | 2 |  |
| **Salvia thyrsiflora* Benth. | 70 | PT |
| *Salvia tiliifolia* Vahl | 40 | MM |
| **Salvia uruapana* Fernald | 15 |  |
| **Salvia vazquezii* Iltis & Ramamoorthy | 6 |  |
