# Supplementary material for: Distribution, richness and conservation of the genus Salvia (Lamiaceae) in the State of Michoacán, Mexico
Source: Biodivers Data J. 2020 Oct 29;8:e56827. doi: 10.3897/BDJ.8.e56827 (PMC7644648; doi:10.3897/BDJ.8.e56827)
Supplement: Supplementary material 2 — Species of Salvia recorded in the State of Michoacán and considered in this study [file bdj-08-e56827-s002.pdf]

Supplementary file 3 –**Ecological niche models of 42 *Salvia* species.** The points on each map show the collecting localities.

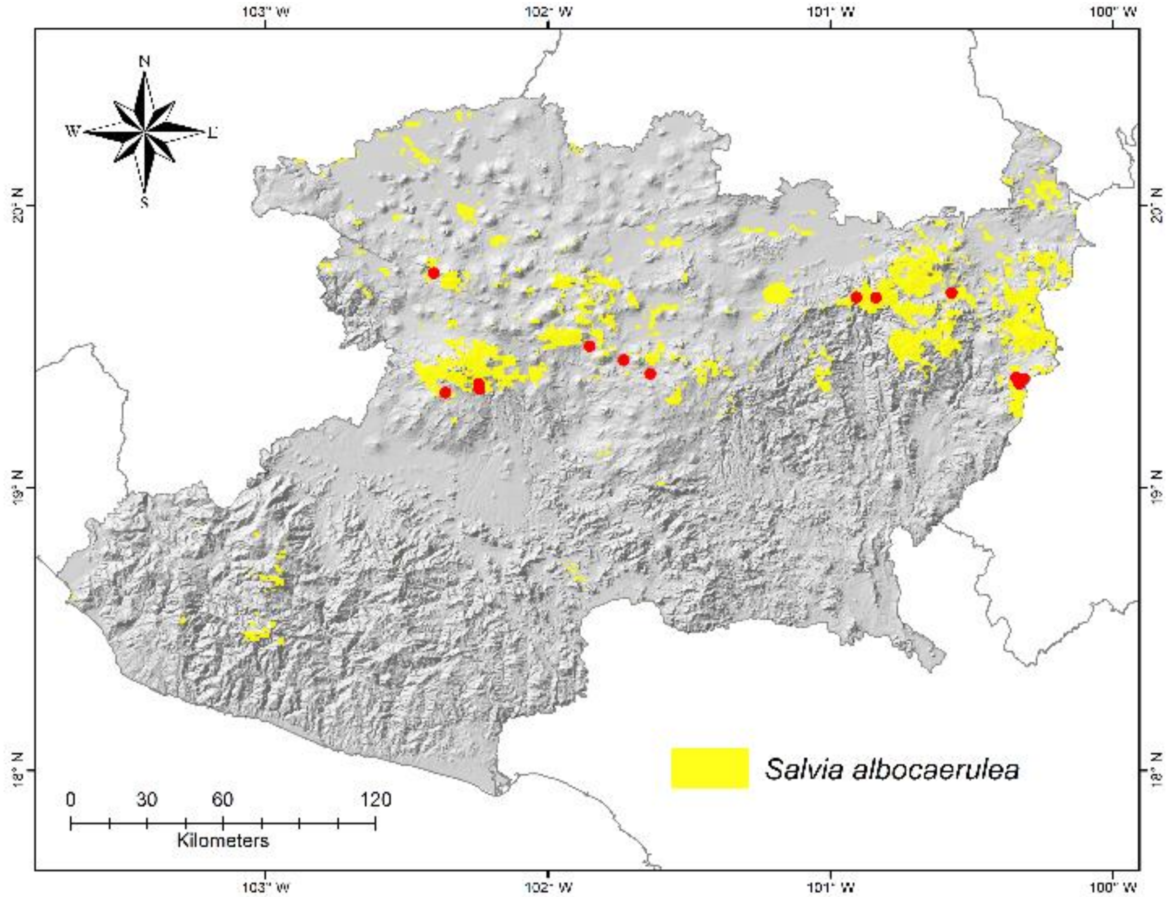

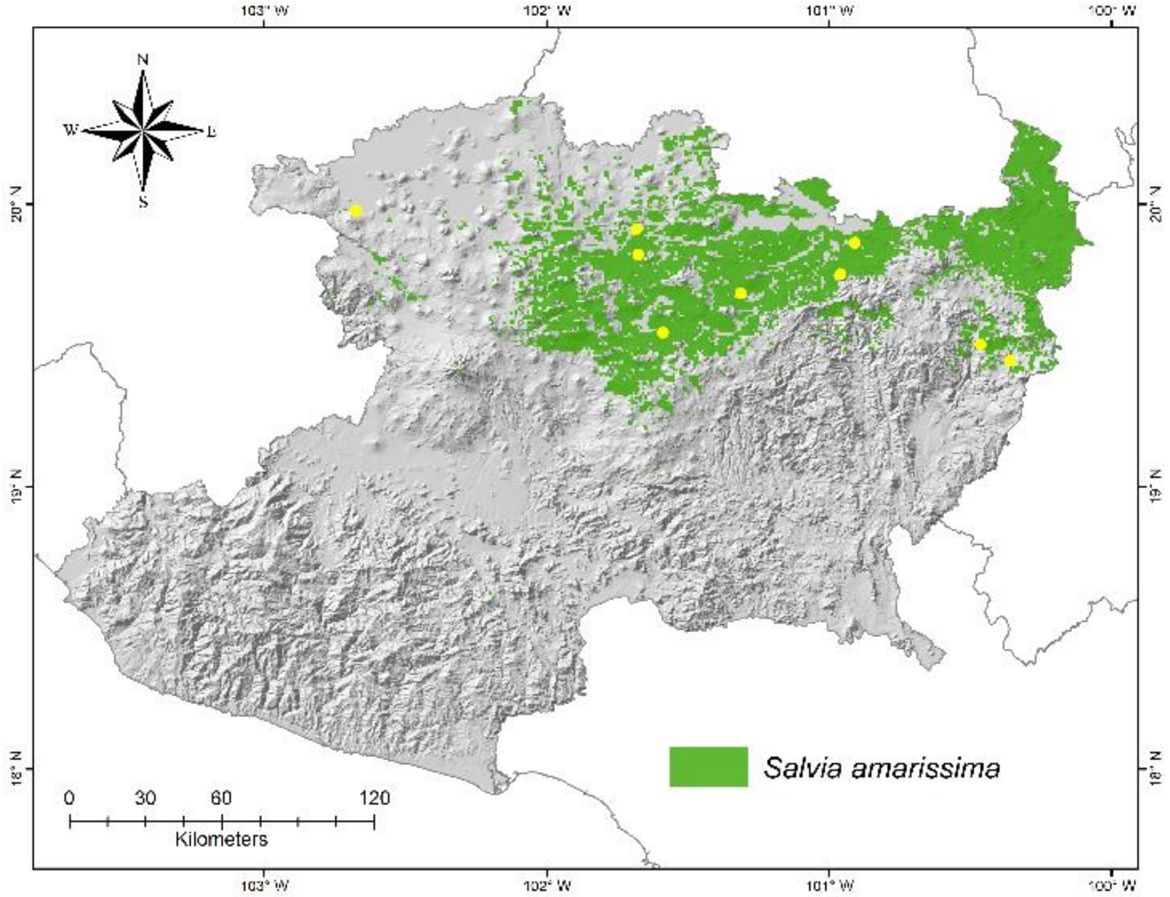

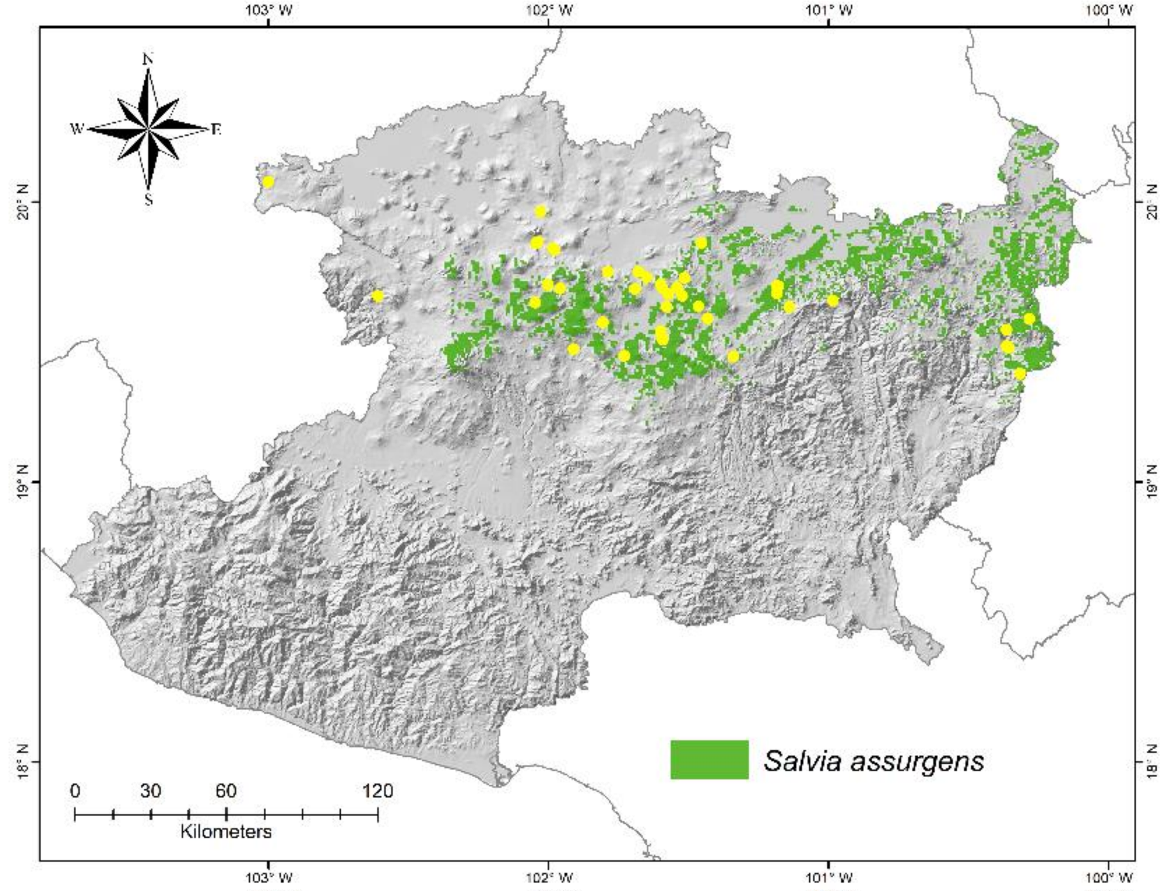
103° W
102° W
101° W
100° W
20° N
19° N
18° N
N
S
E
W
Salvia assurgens
0
30
60
120
Kilometers

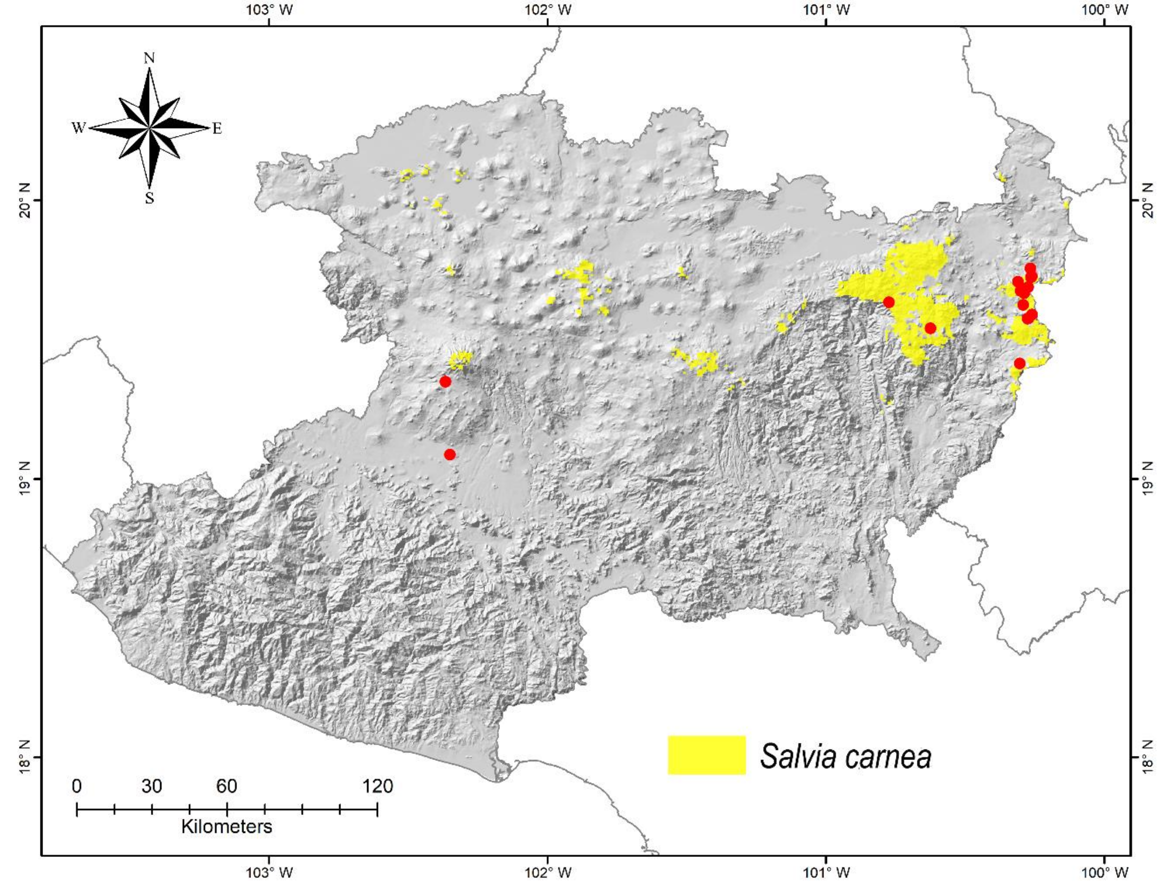
103° W
102° W
101° W
100° W
20° N
19° N
18° N
N
S
E
W
Salvia carnea
0
30
60
120
Kilometers

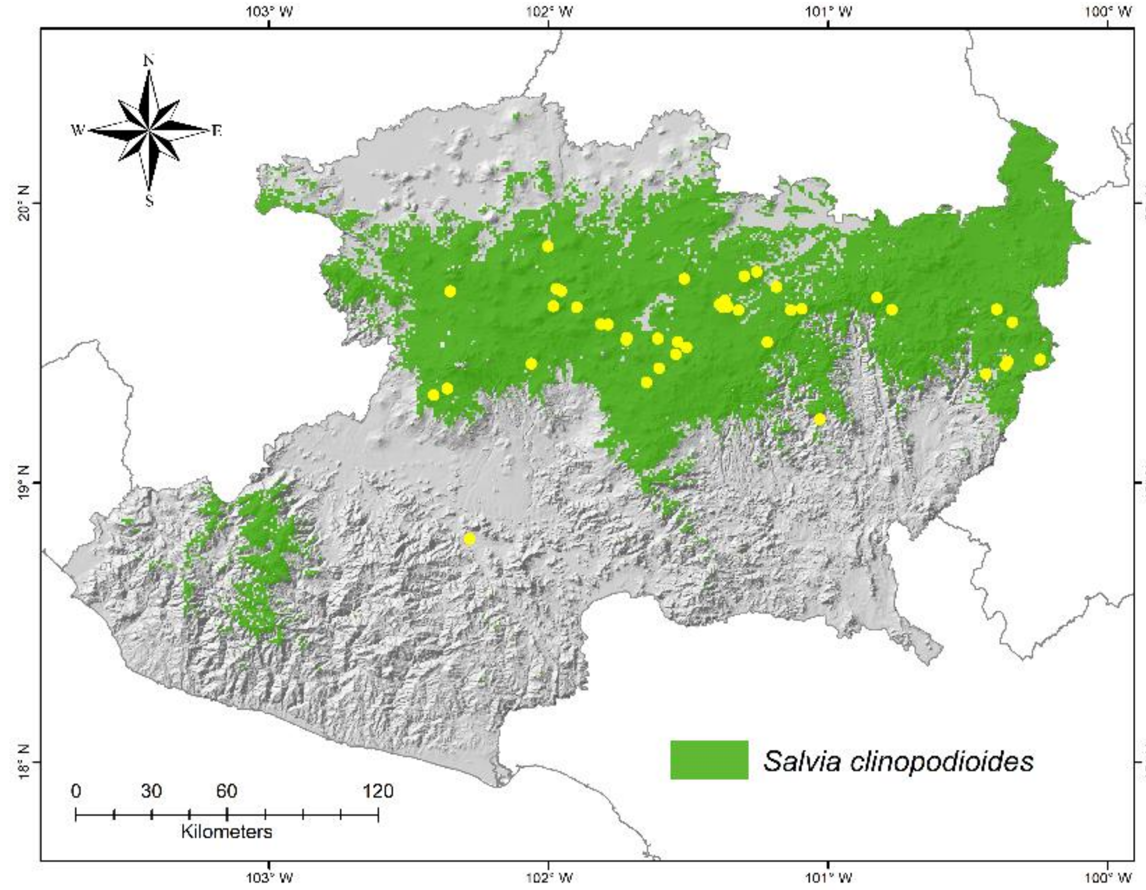
103° W
102° W
101° W
100° W
20° N
19° N
18° N
N
W
E
S
Salvia clinopodioides
0
30
60
120
Kilometers

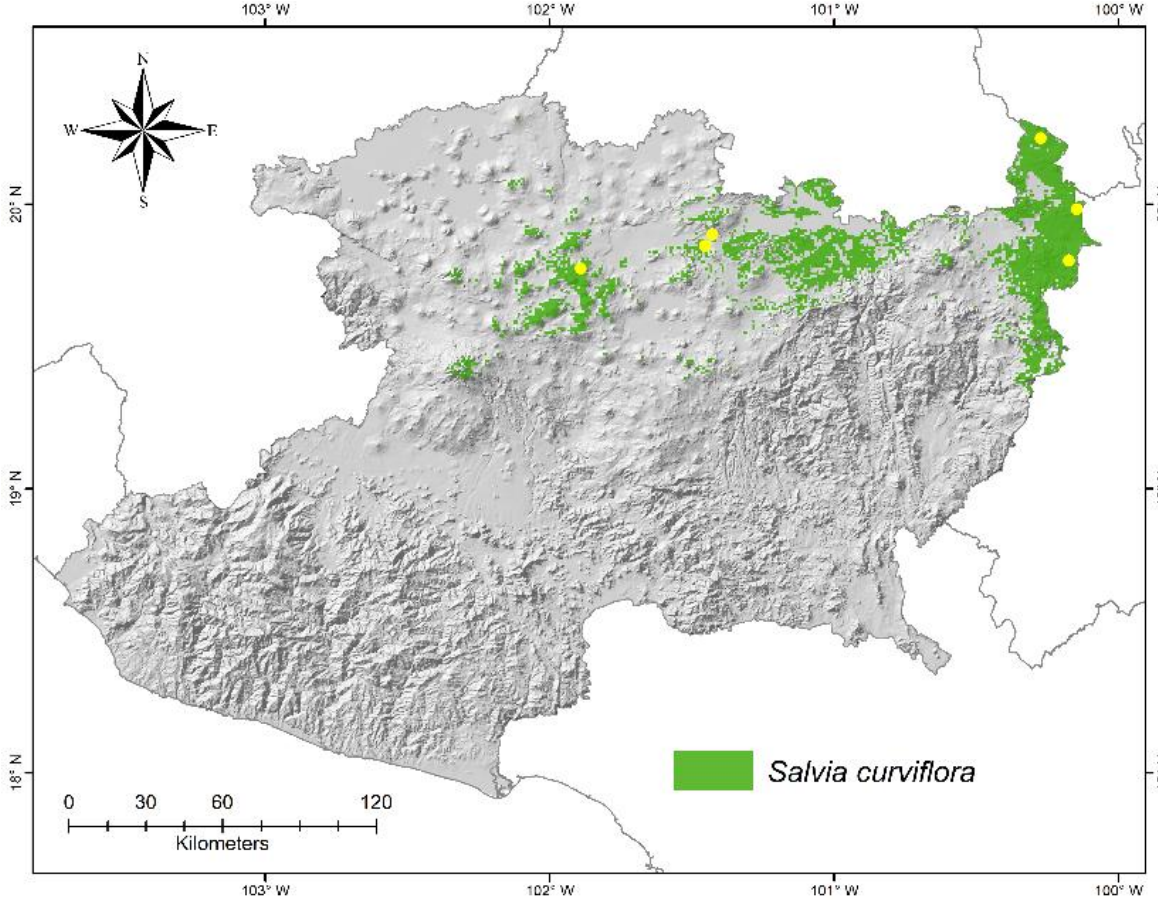
103° W
102° W
101° W
100° W
20° N
19° N
18° N
N
W
E
S
Salvia curviflora
0
30
60
120
Kilometers

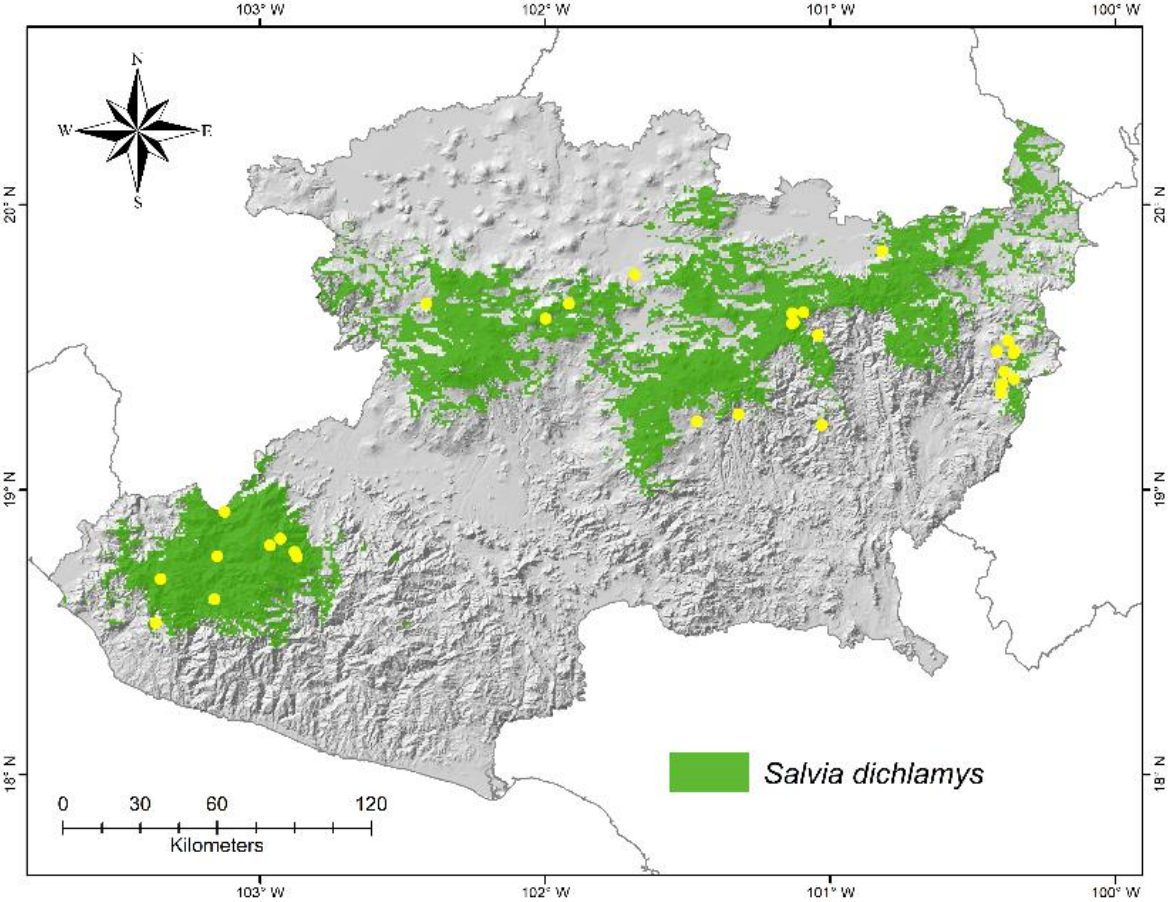
103° W
102° W
101° W
100° W
20° N
19° N
18° N
N
S
W
E
Salvia dichlamys
0
30
60
120
Kilometers

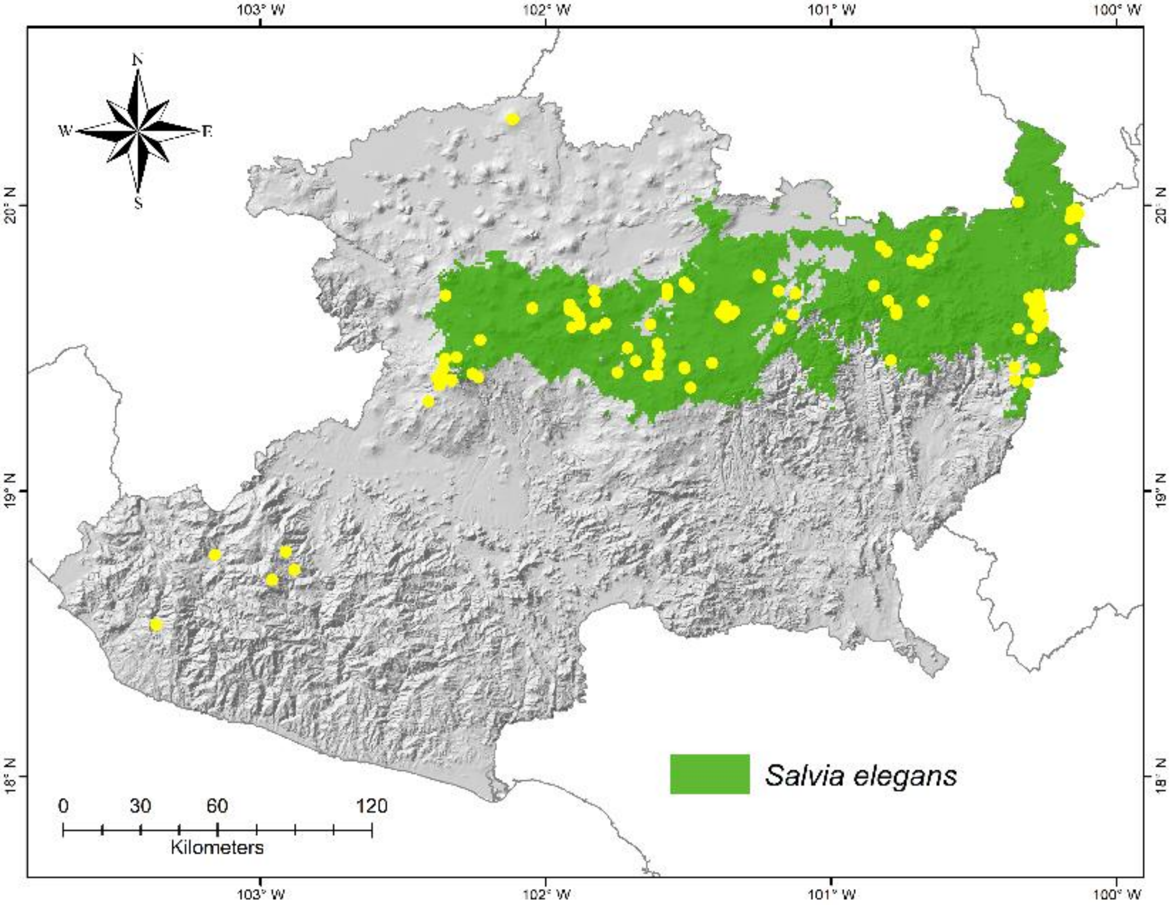
103° W
102° W
101° W
100° W
20° N
19° N
18° N
N
S
W
E
Salvia elegans
0
30
60
120
Kilometers

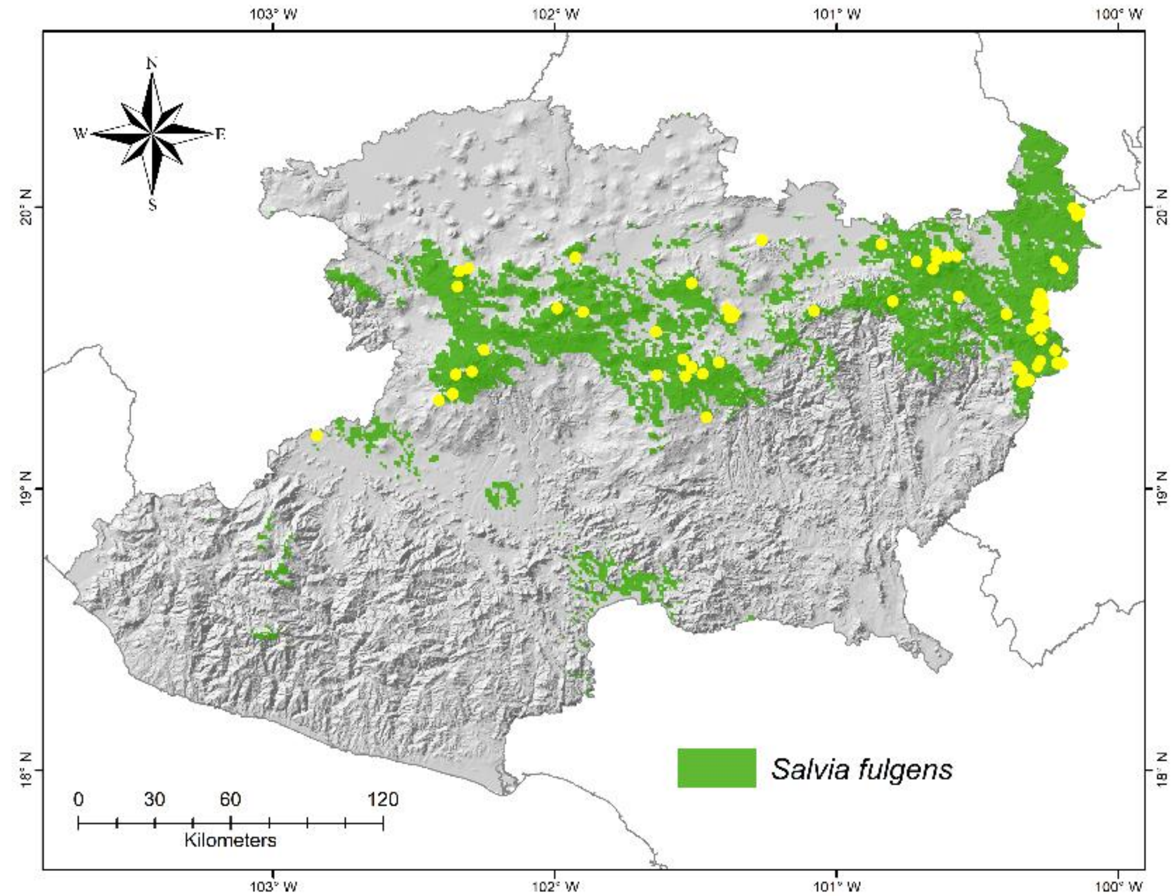
103° W
102° W
101° W
100° W
20° N
19° N
18° N
N
S
E
W
Salvia fulgens
0
30
60
120
Kilometers

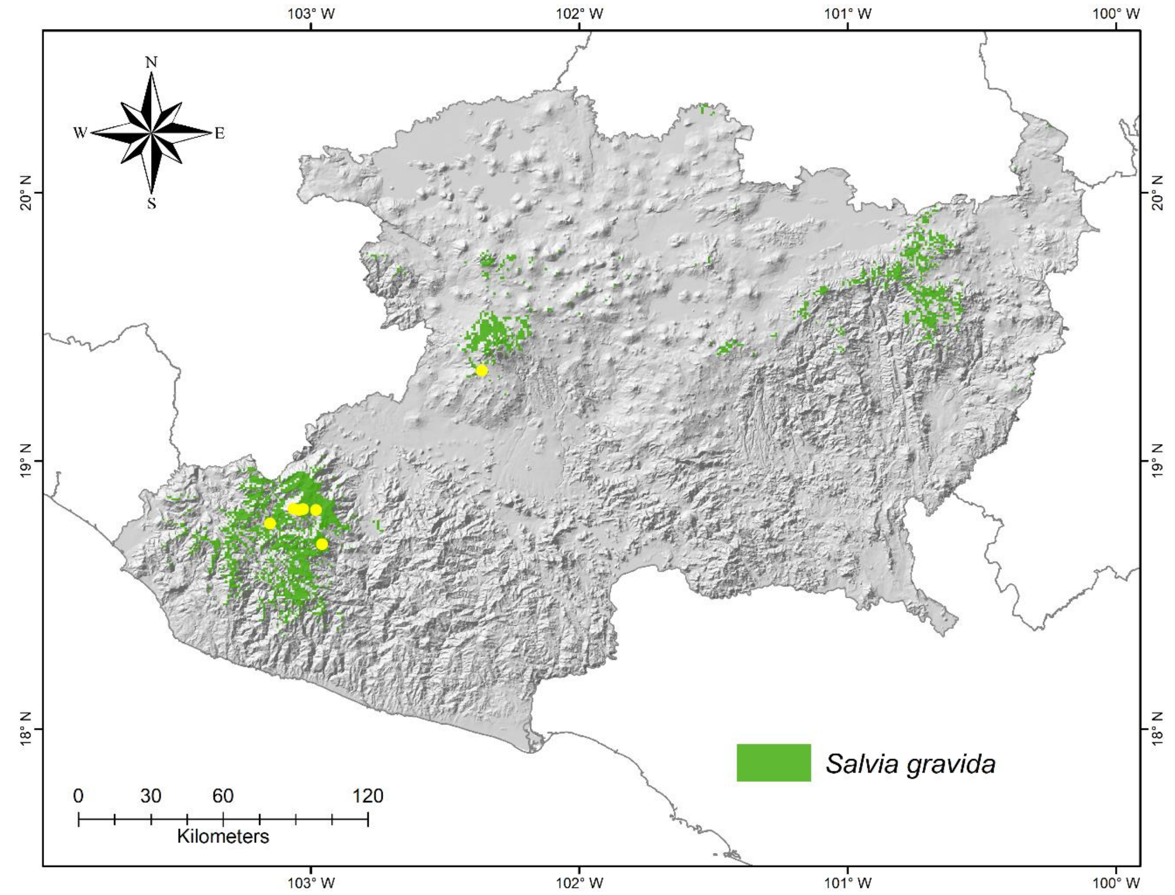
103° W
102° W
101° W
100° W
20° N
19° N
18° N
N
S
E
W
Salvia gravida
0
30
60
120
Kilometers

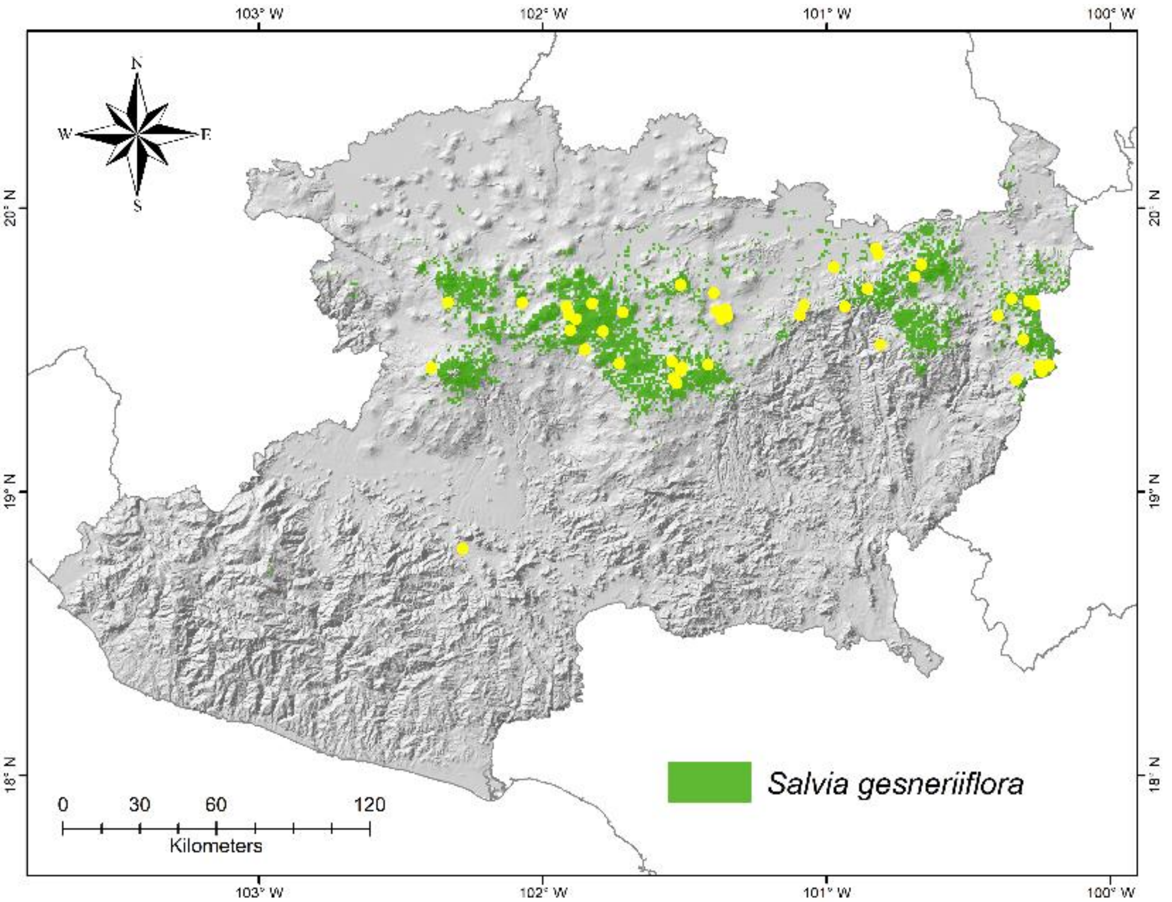
103° W
102° W
101° W
100° W
20° N
19° N
18° N
N
S
W
E
Salvia gesneriiflora
0
30
60
120
Kilometers

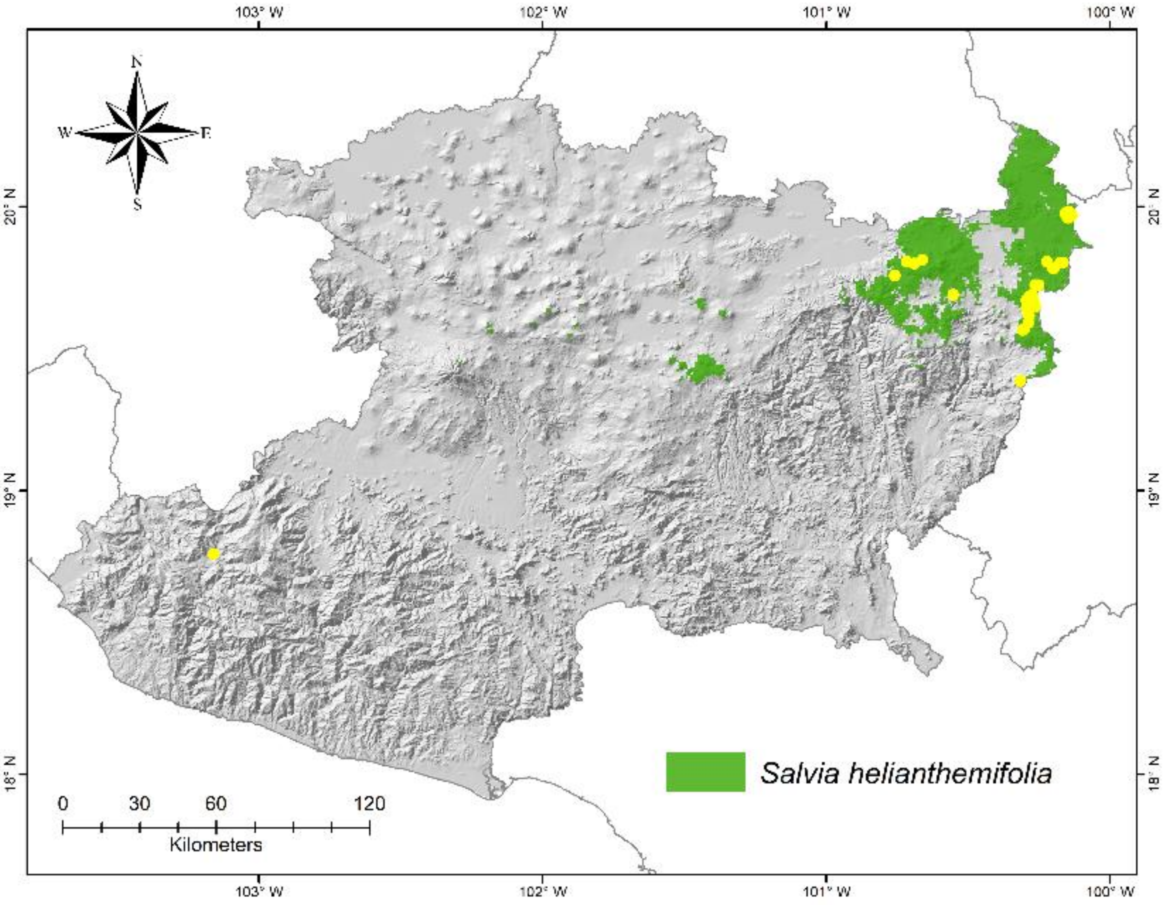
103° W
102° W
101° W
100° W
20° N
19° N
18° N
N
S
W
E
Salvia helianthemifolia
0
30
60
120
Kilometers

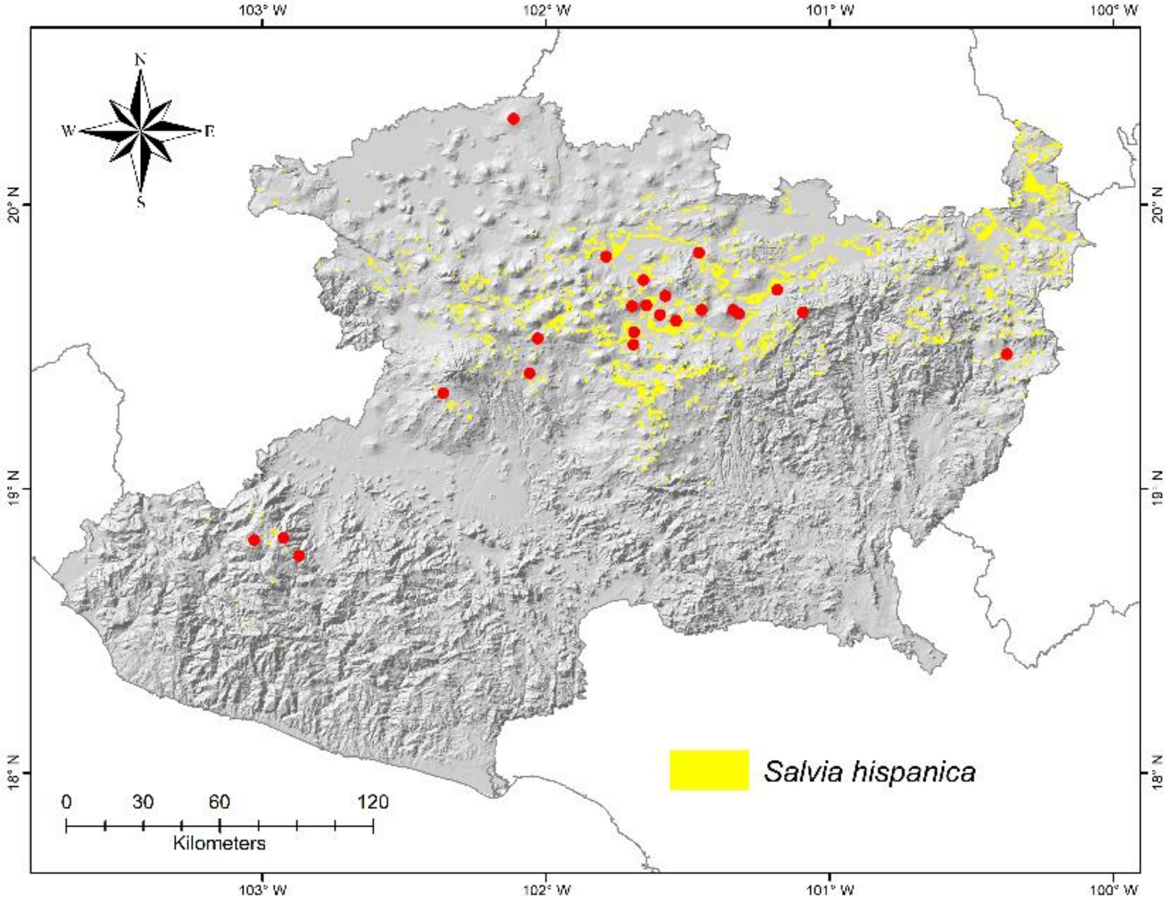

103° W
102° W
101° W
100° W
20° N
19° N
18° N
N
S
W
E
Salvia hispanica
0
30
60
120
Kilometers

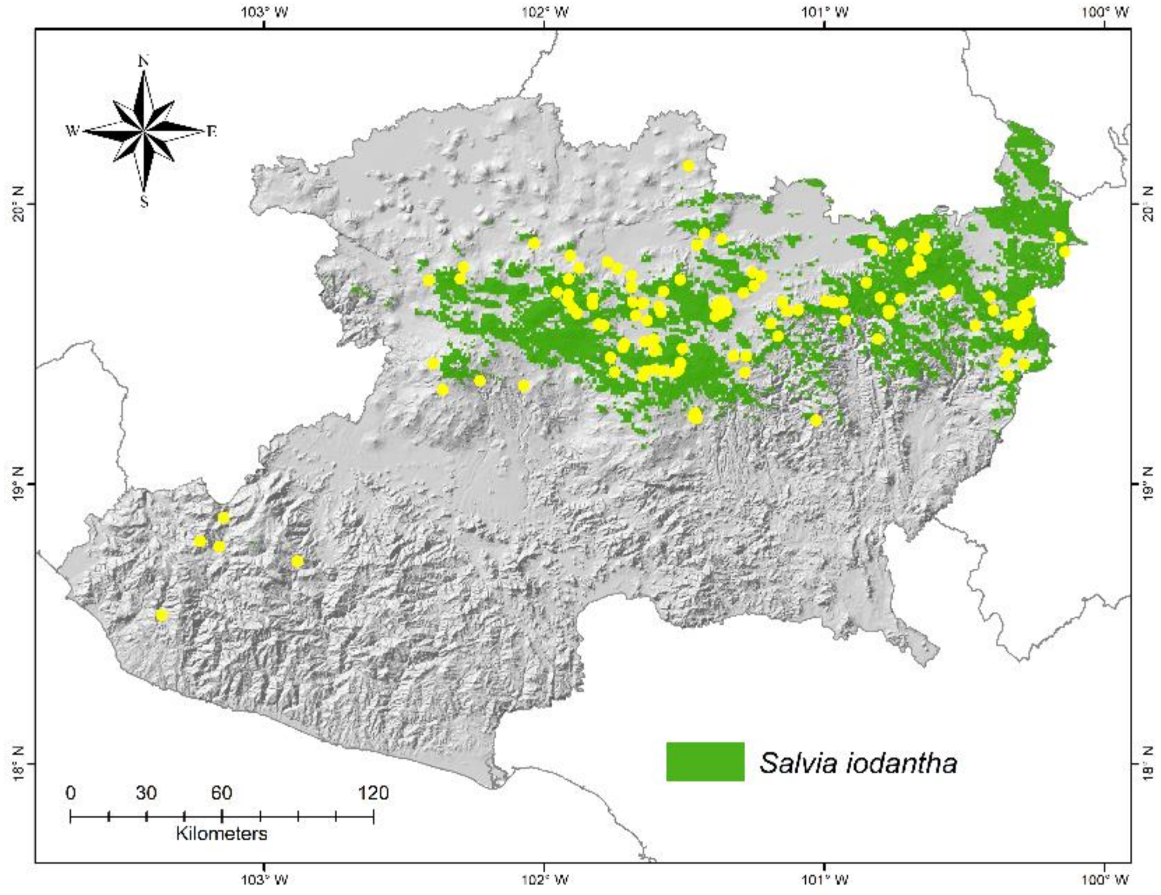

103° W
102° W
101° W
100° W
20° N
19° N
18° N
N
S
W
E
Salvia iodantha
0
30
60
120
Kilometers

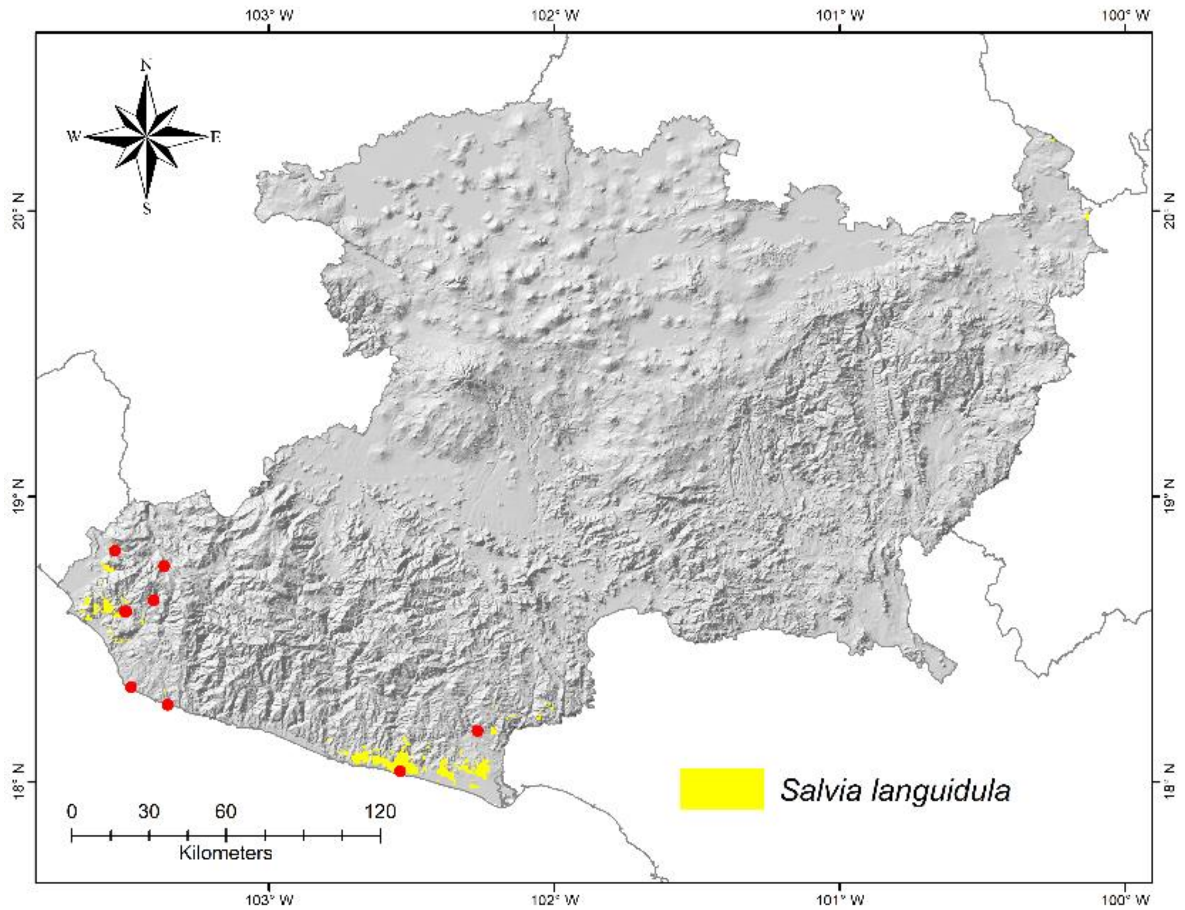

103° W
102° W
101° W
100° W
20° N
19° N
18° N
N
S
W
E
Salvia languidula
0
30
60
120
Kilometers

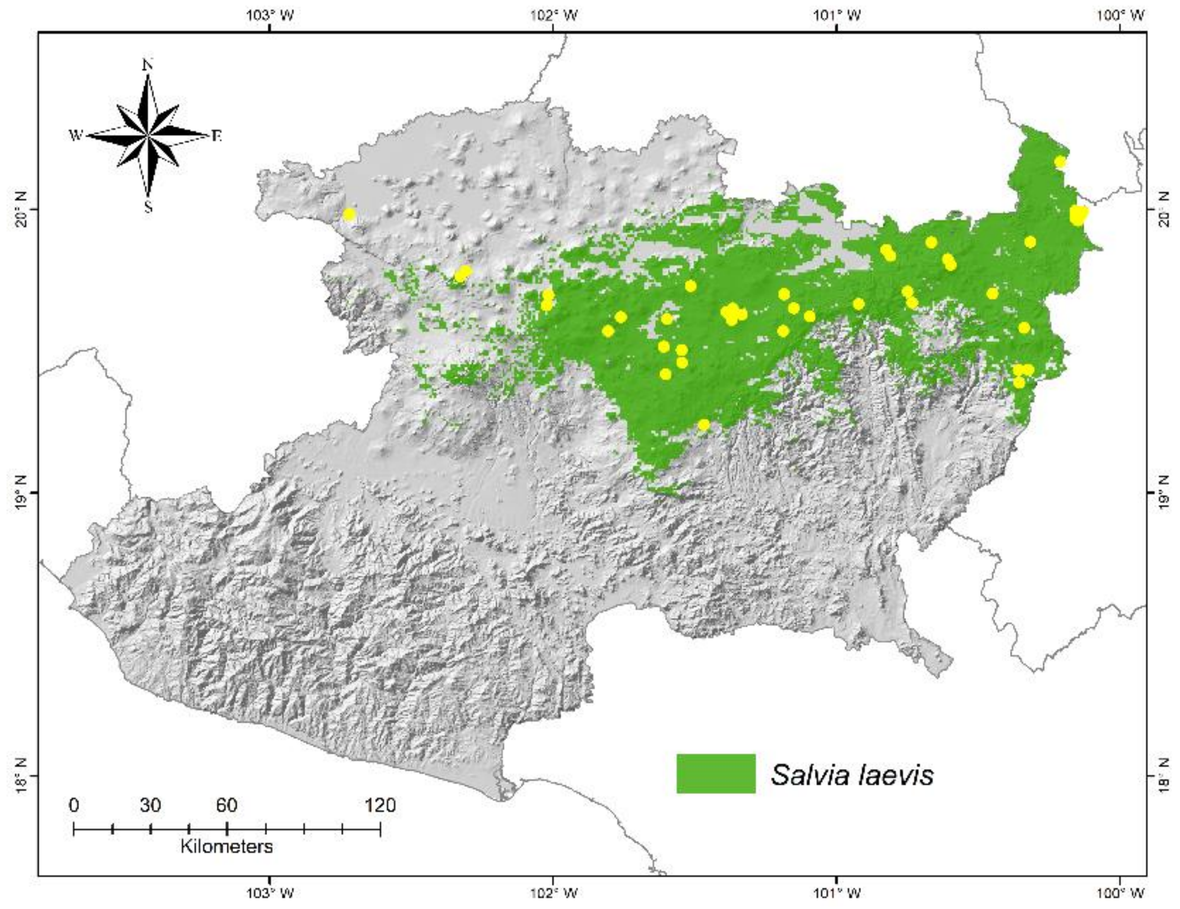

103° W
102° W
101° W
100° W
20° N
19° N
18° N
N
S
W
E
Salvia laevis
0
30
60
120
Kilometers

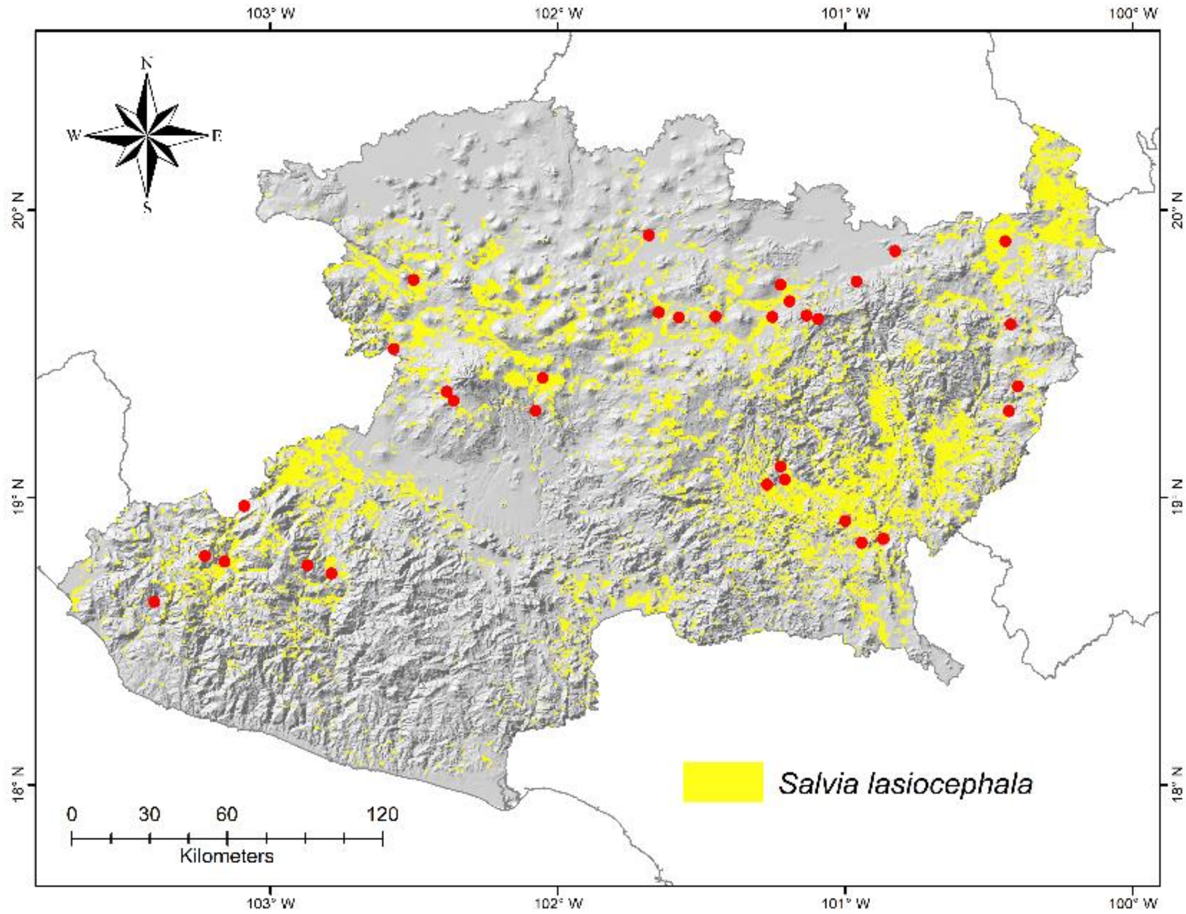

103° W
102° W
101° W
100° W
20° N
19° N
18° N
N
S
W
E
0
30
60
120
Kilometers
Salvia lasiocephala

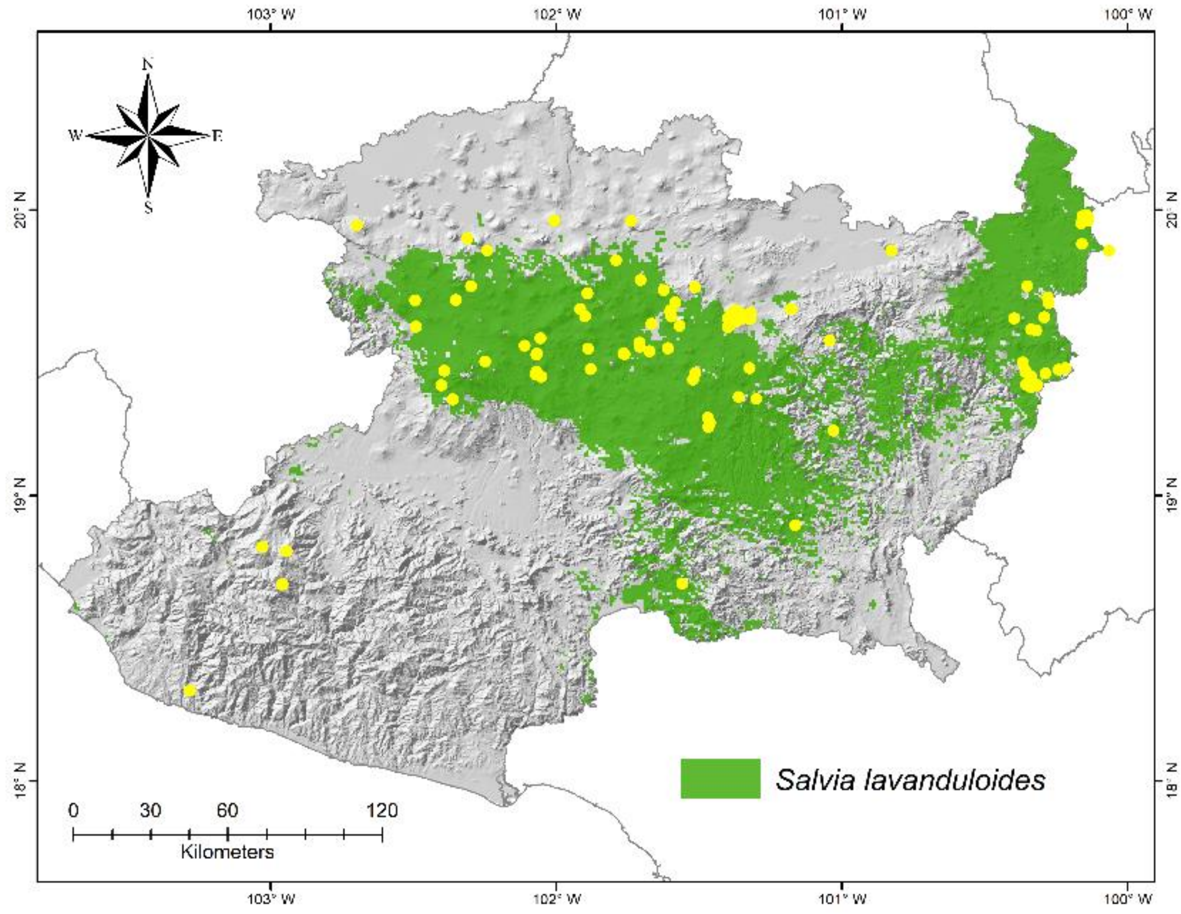

103° W
102° W
101° W
100° W
20° N
19° N
18° N
N
S
W
E
0
30
60
120
Kilometers
Salvia lavanduloides

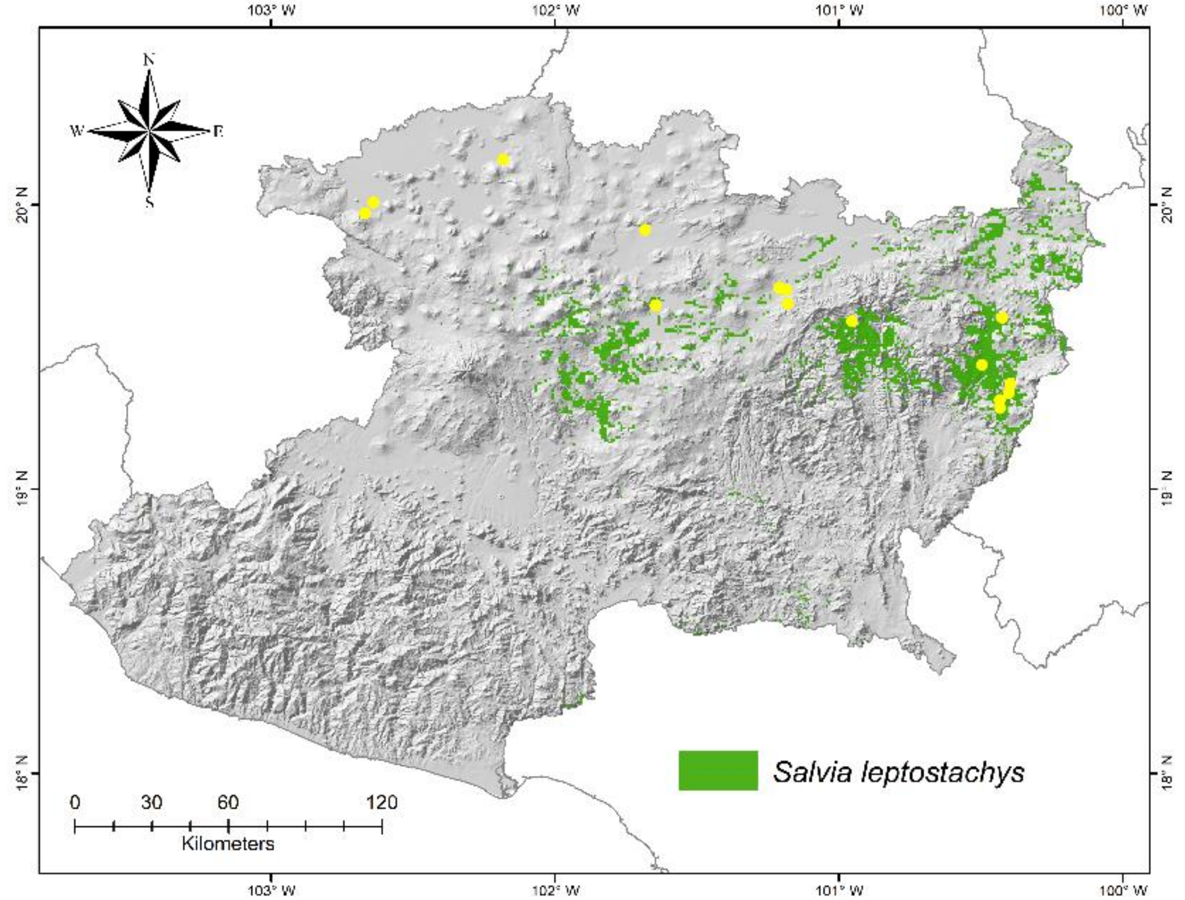
103° W
102° W
101° W
100° W
20° N
19° N
18° N
N
S
W
E
Salvia leptostachys
0
30
60
120
Kilometers

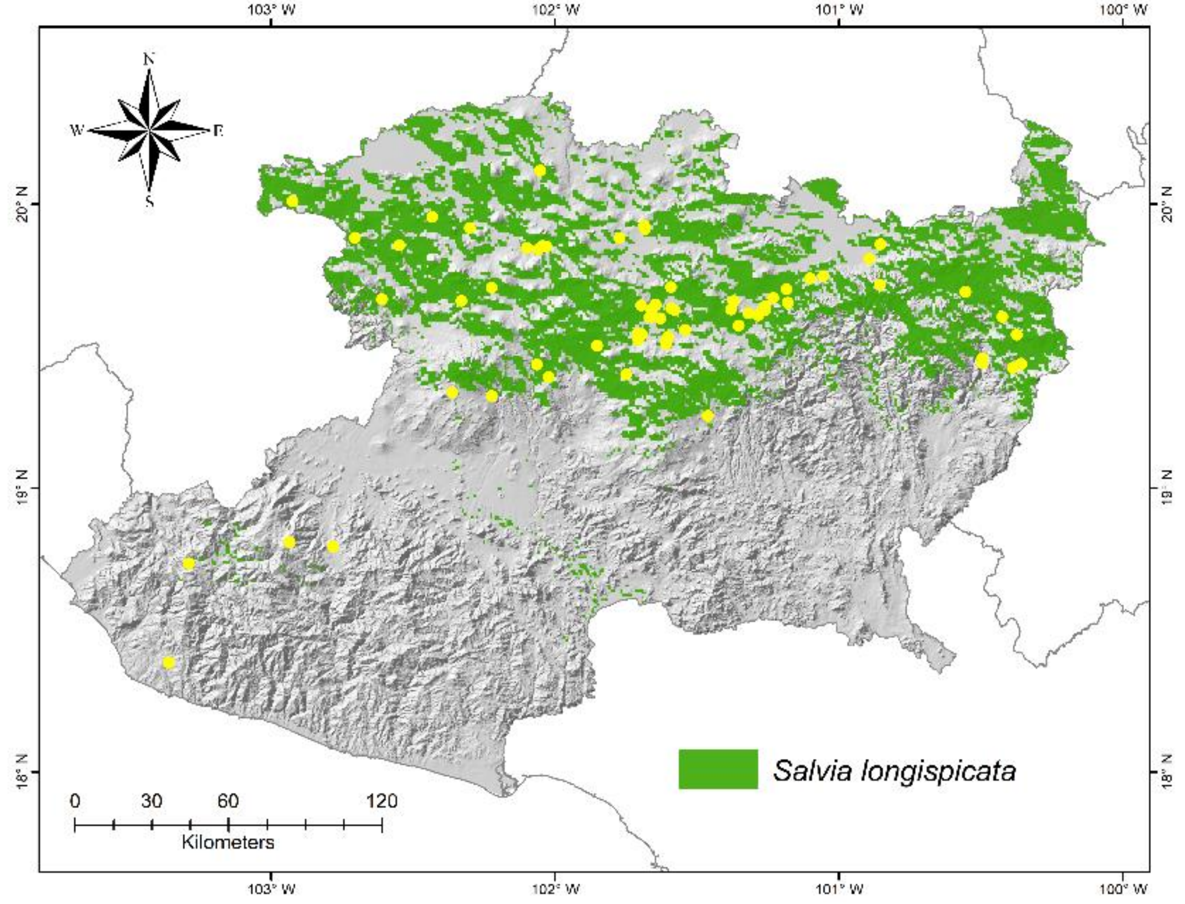
103° W
102° W
101° W
100° W
20° N
19° N
18° N
N
S
W
E
Salvia longispicata
0
30
60
120
Kilometers

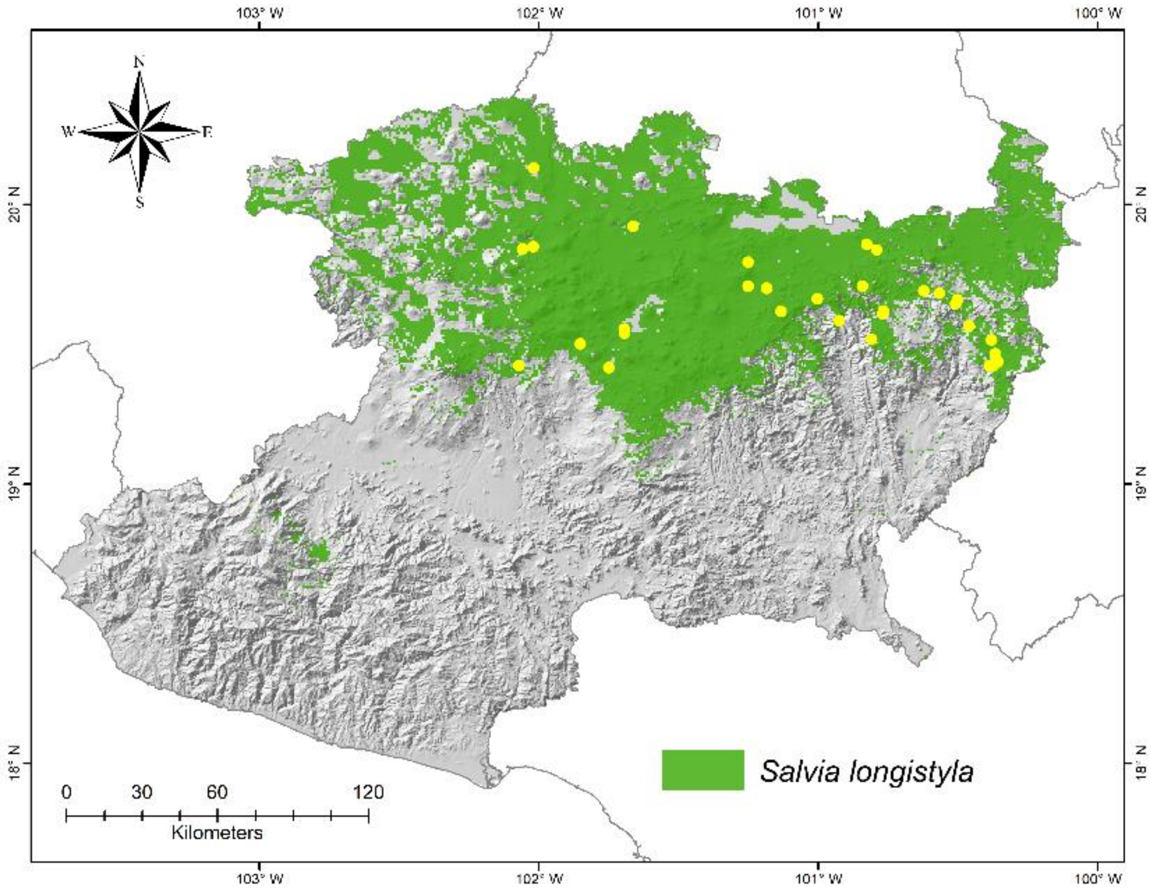
103° W
102° W
101° W
100° W
20° N
19° N
18° N
N
S
W
E
Salvia longistyla
0
30
60
120
Kilometers

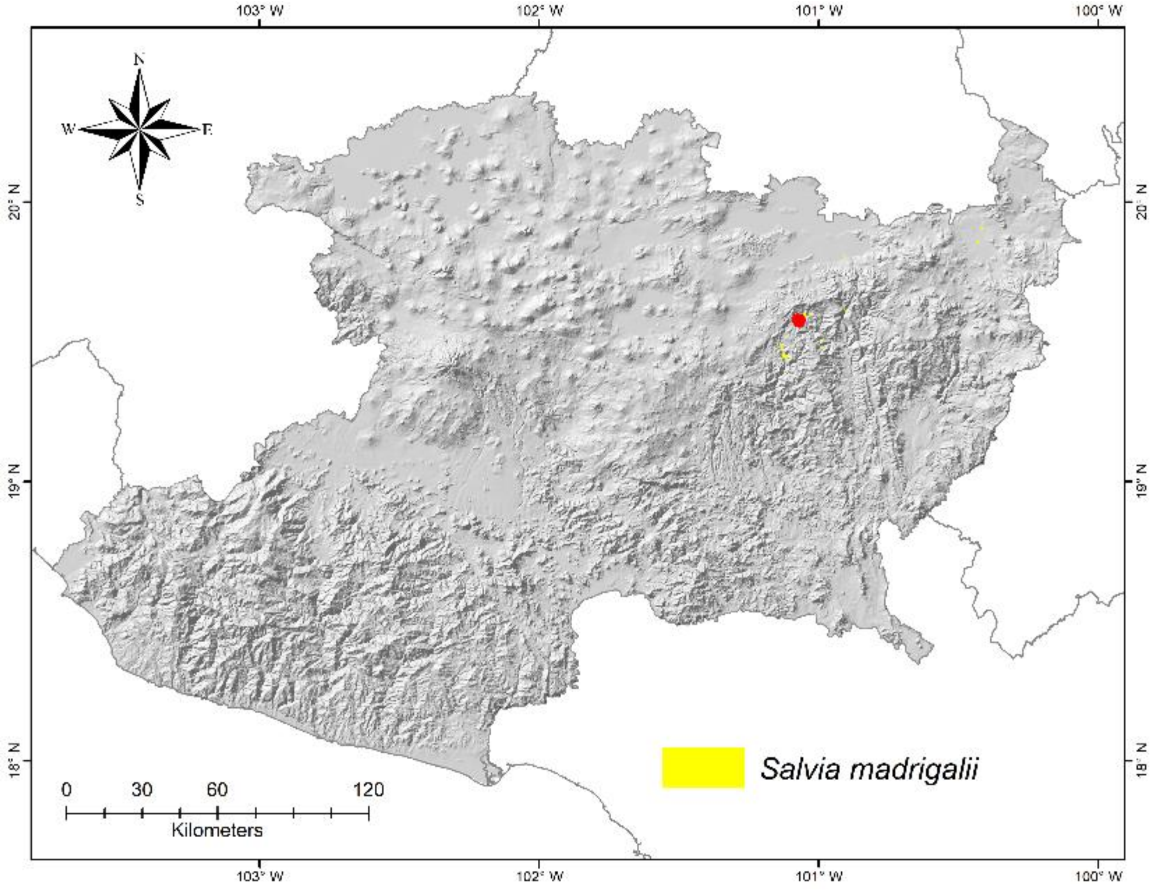
103° W
102° W
101° W
100° W
20° N
19° N
18° N
N
S
W
E
Salvia madrigalii
0
30
60
120
Kilometers

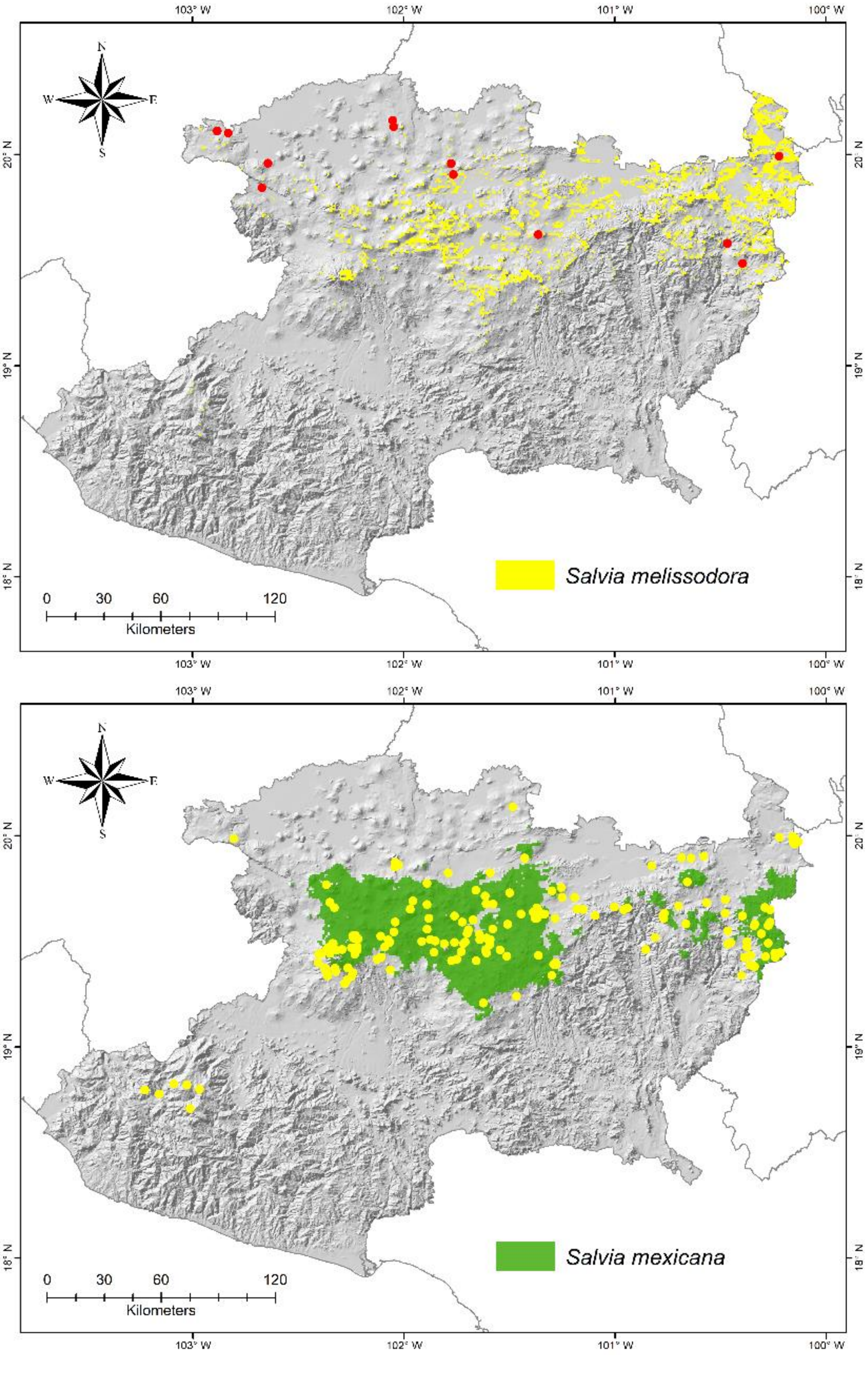
103° W
102° W
101° W
100° W
20° N
19° N
18° N
N
S
W
E
Salvia melissodora
0
30
60
120
Kilometers
Salvia mexicana

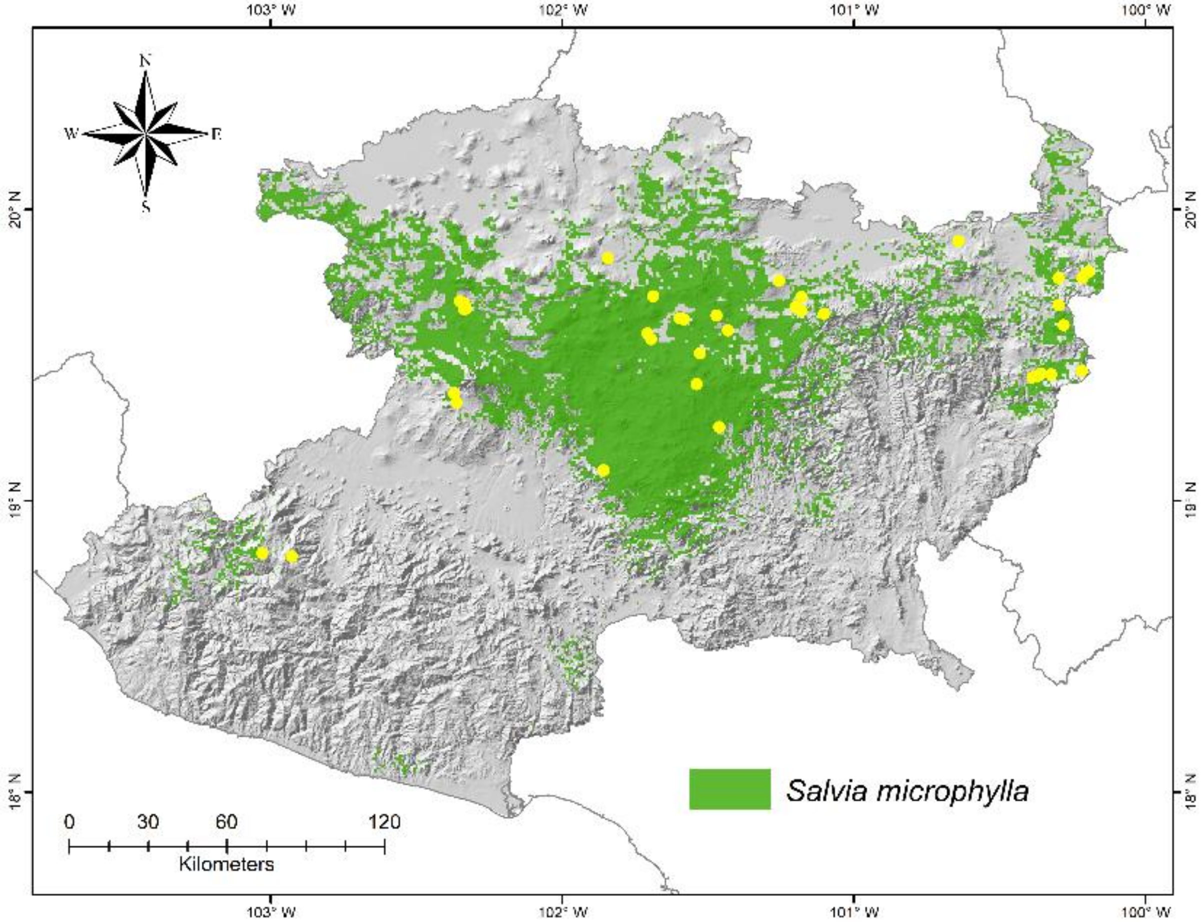
103° W
102° W
101° W
100° W
20° N
19° N
18° N
N
S
W
E
Salvia microphylla
0
30
60
120
Kilometers

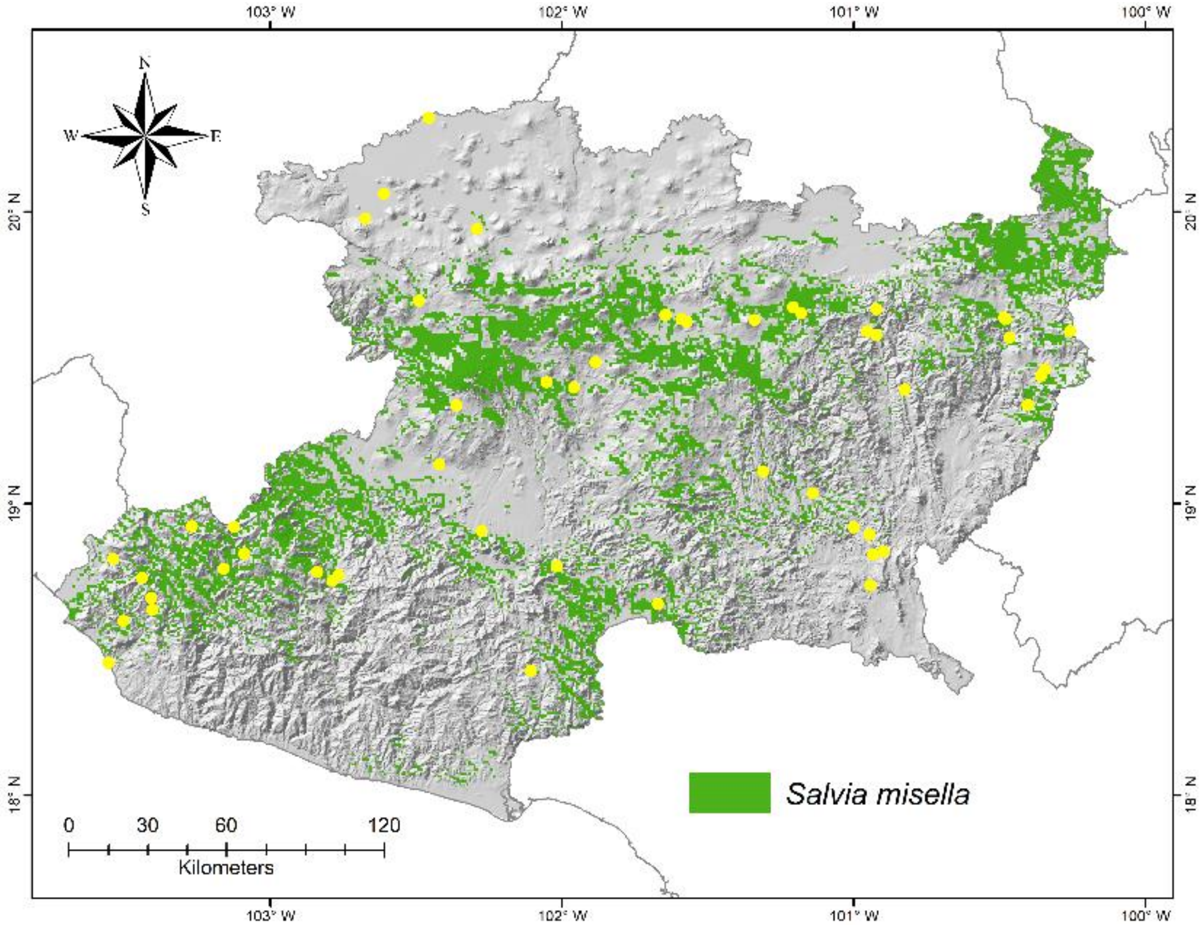
103° W
102° W
101° W
100° W
20° N
19° N
18° N
N
S
W
E
Salvia misella
0
30
60
120
Kilometers

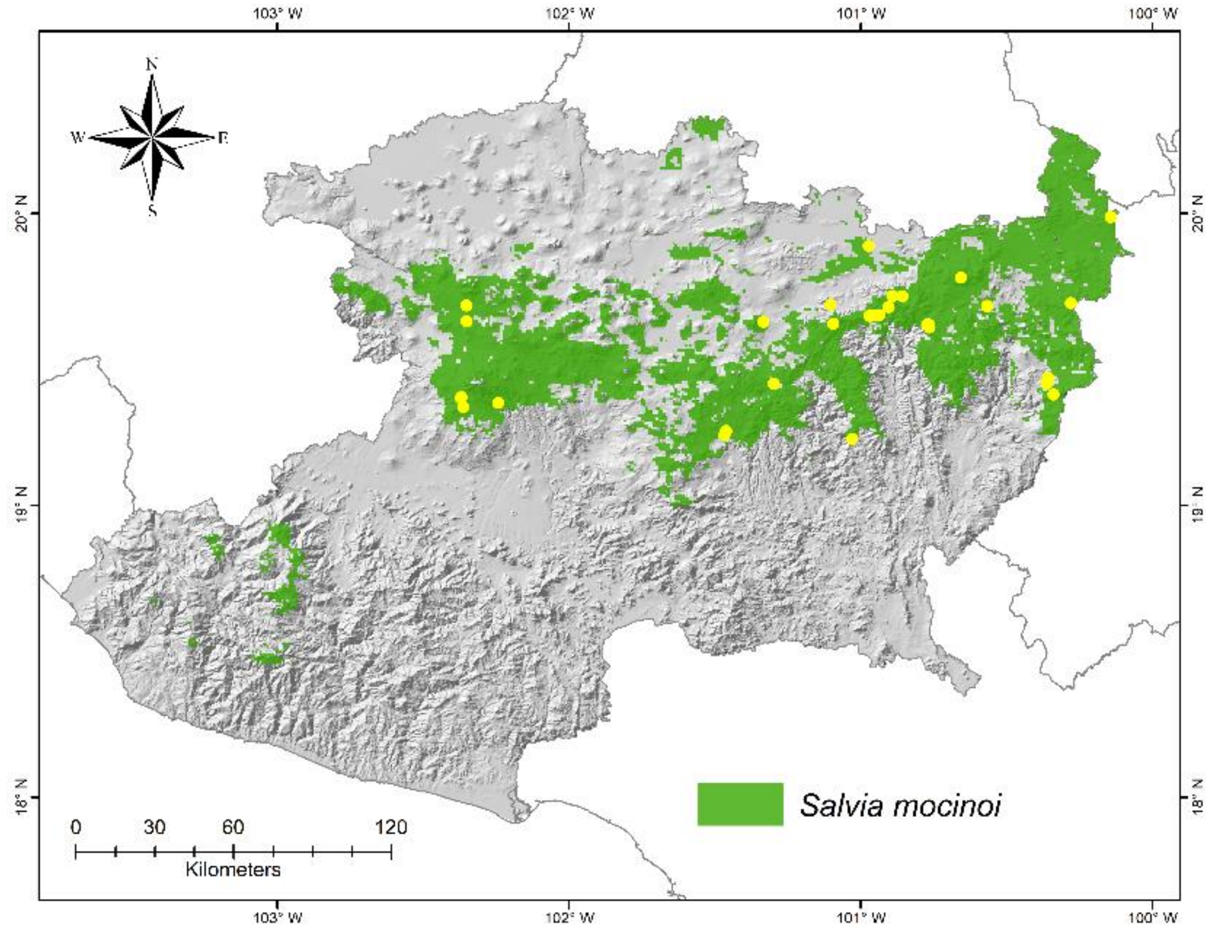

103° W
102° W
101° W
100° W
20° N
19° N
18° N
N
S
W
E
0
30
60
120
Kilometers
Salvia mocinoi

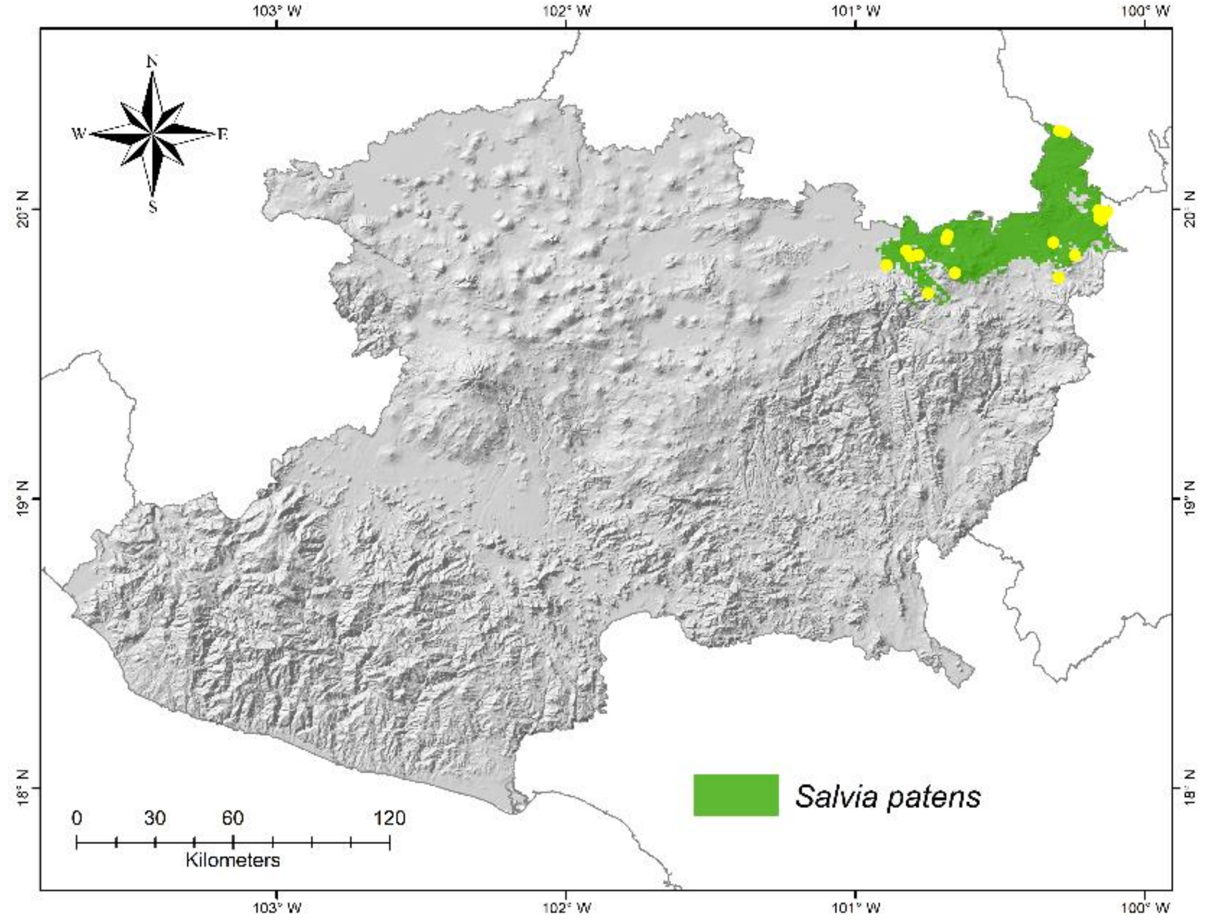

103° W
102° W
101° W
100° W
20° N
19° N
18° N
N
S
W
E
0
30
60
120
Kilometers
Salvia patens

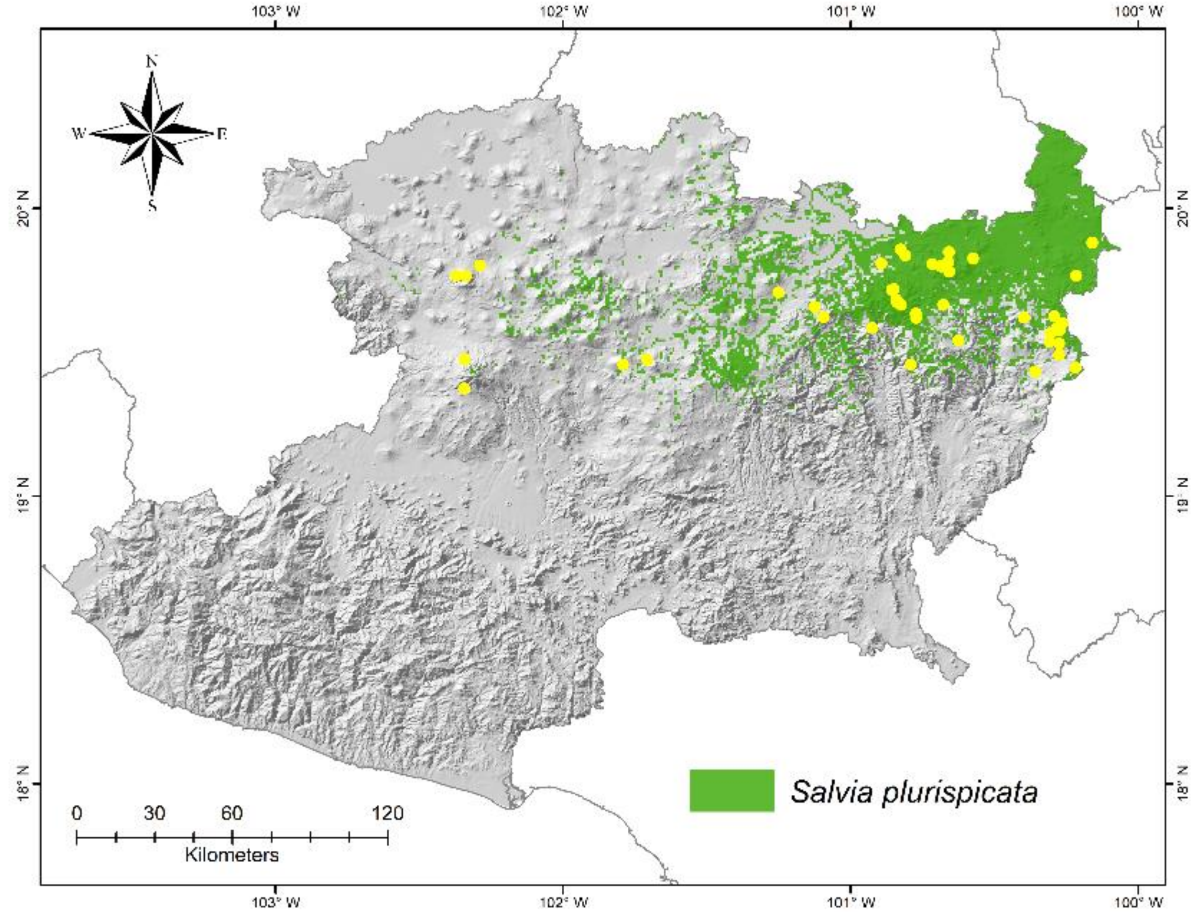
103° W
102° W
101° W
100° W
20° N
19° N
18° N
N
S
W
E
Salvia plurispicata
0
30
60
120
Kilometers

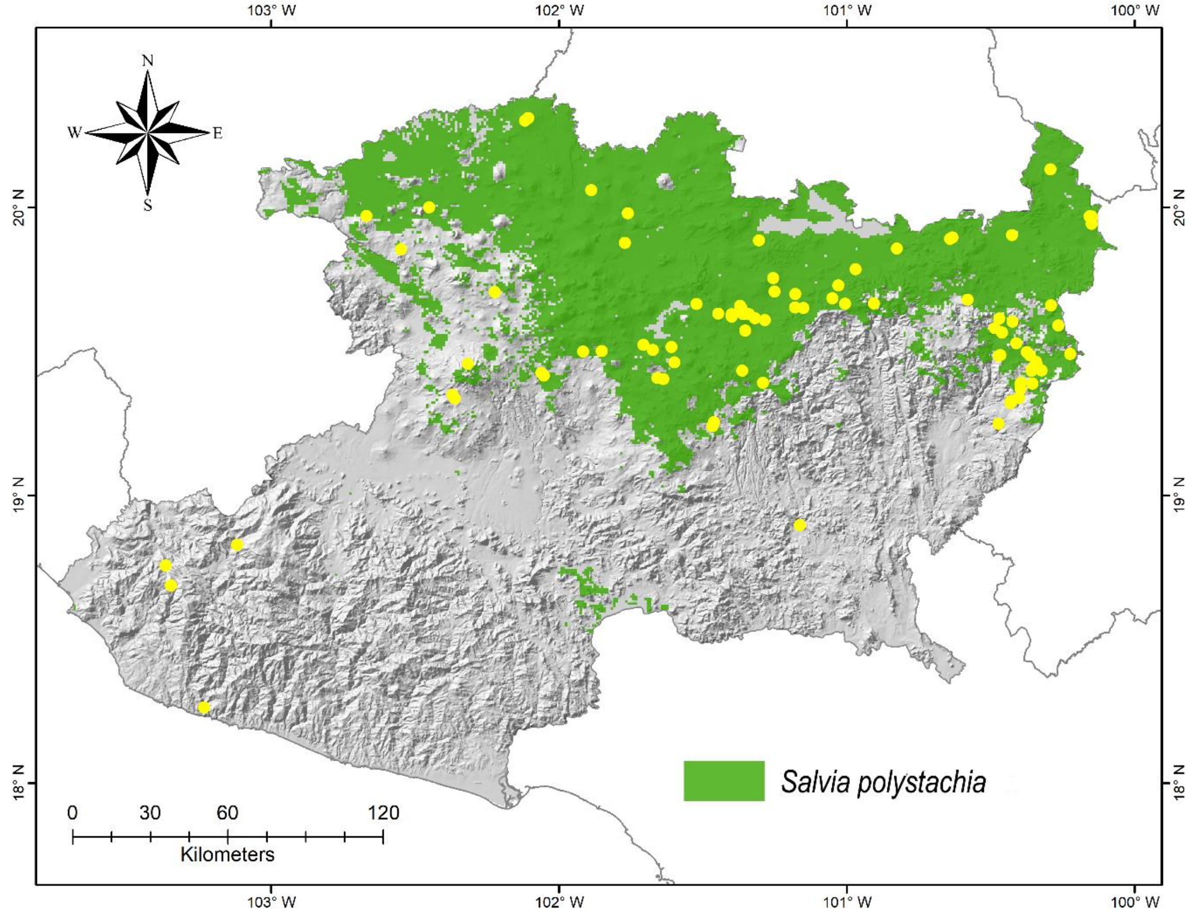
103° W
102° W
101° W
100° W
20° N
19° N
18° N
N
S
W
E
Salvia polystachia
0
30
60
120
Kilometers

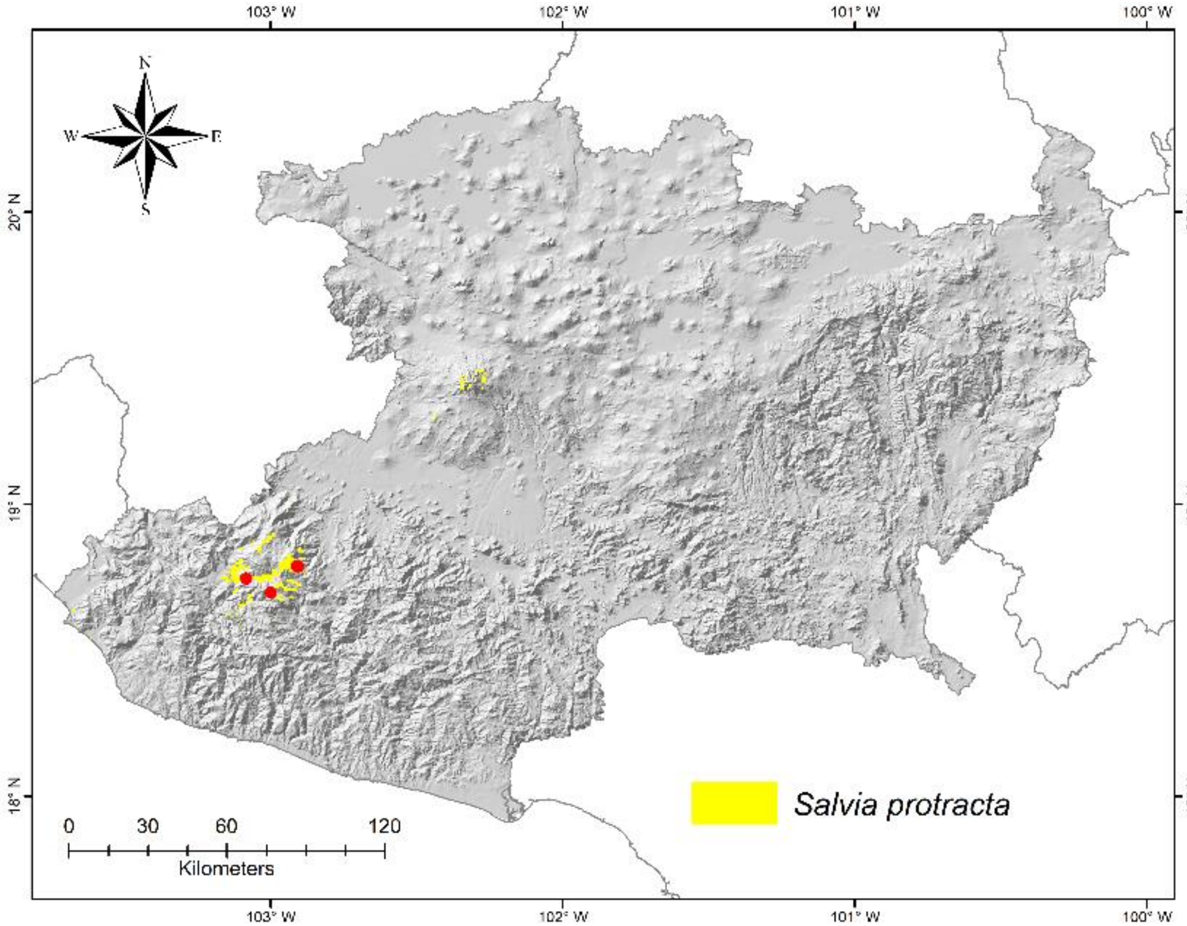
103° W
102° W
101° W
100° W
N
W
E
S
20° N
19° N
18° N
0
30
60
120
Kilometers
Salvia protracta

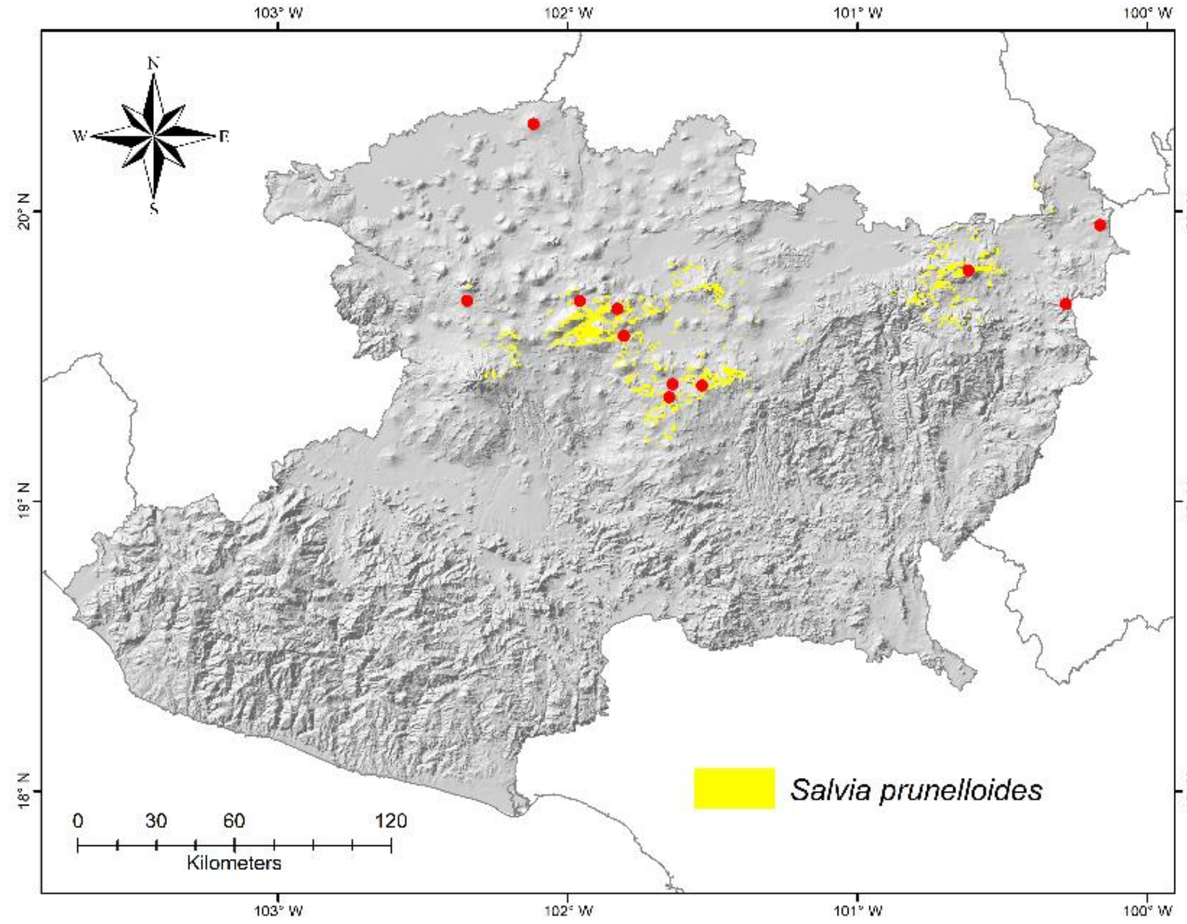
103° W
102° W
101° W
100° W
N
W
E
S
20° N
19° N
18° N
0
30
60
120
Kilometers
Salvia prunelloides

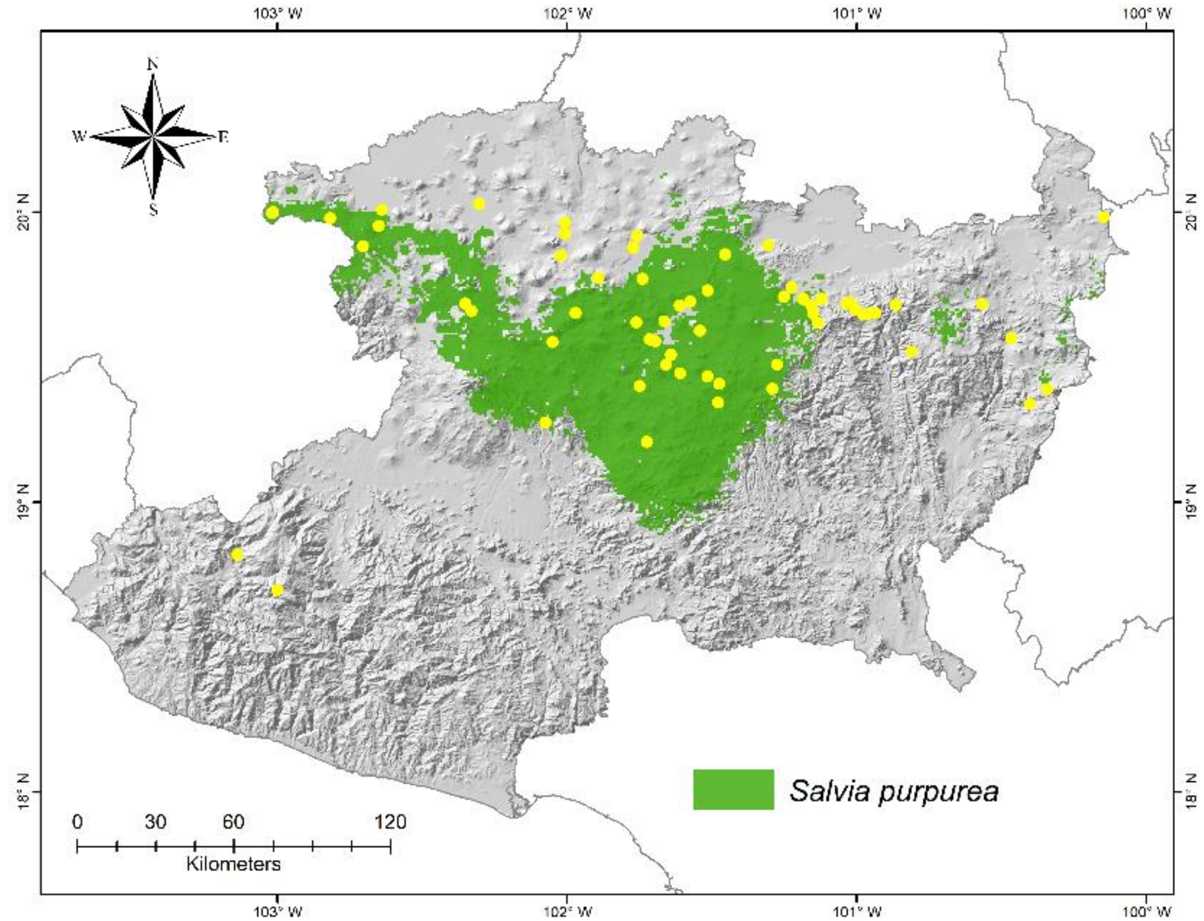
103° W
102° W
101° W
100° W
20° N
19° N
18° N
N
S
W
E
0
30
60
120
Kilometers
Salvia purpurea

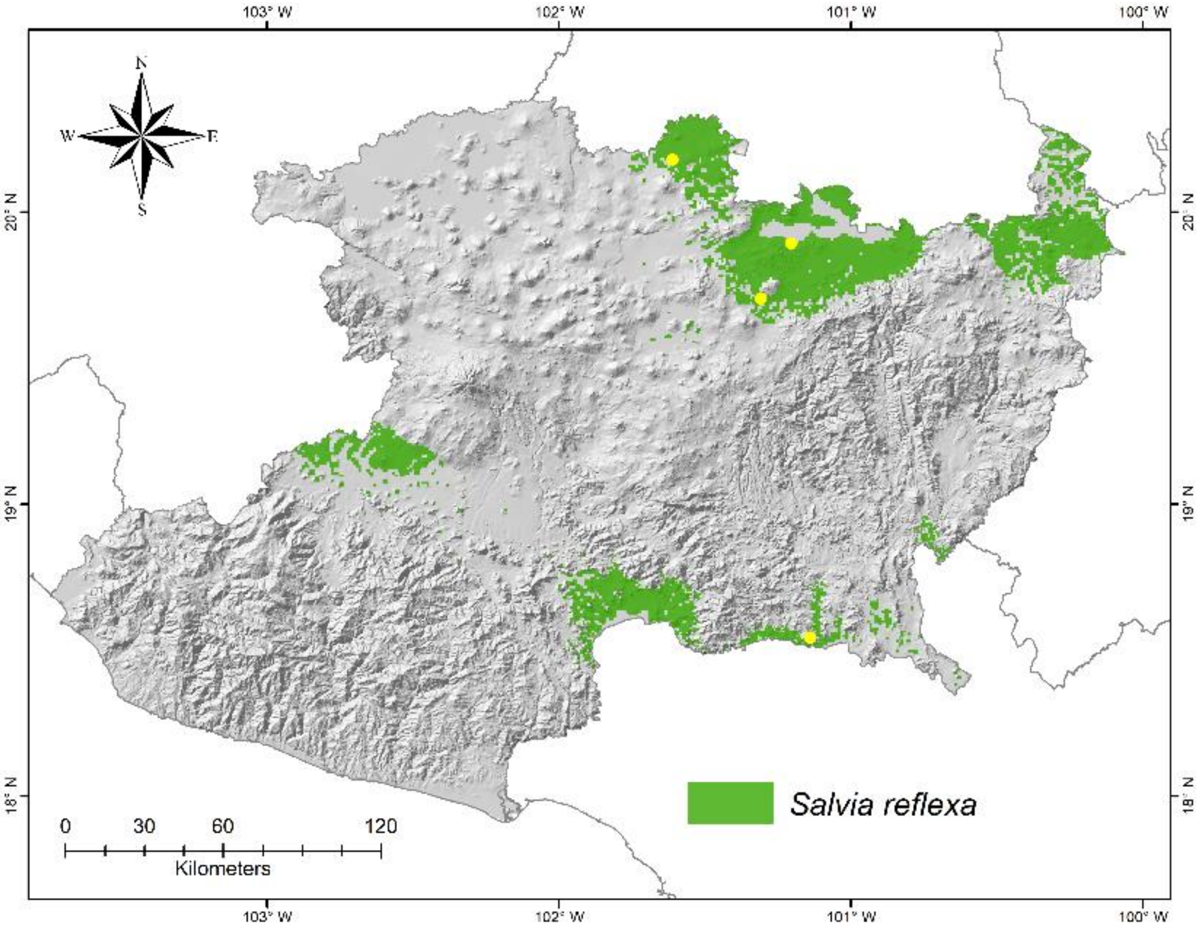
103° W
102° W
101° W
100° W
20° N
19° N
18° N
N
S
W
E
0
30
60
120
Kilometers
Salvia reflexa

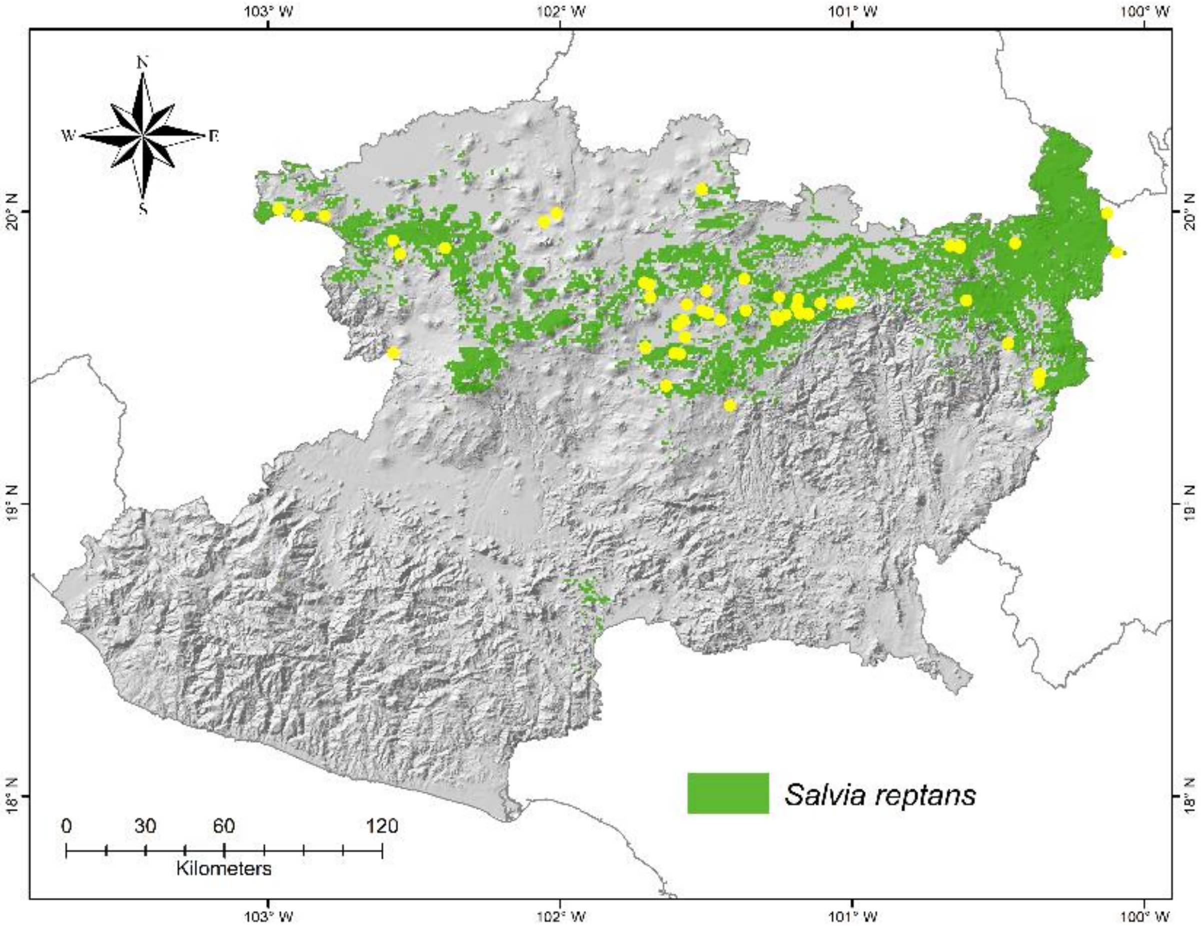
103° W
102° W
101° W
100° W
20° N
19° N
18° N
N
S
W
E
Salvia reptans
0
30
60
120
Kilometers

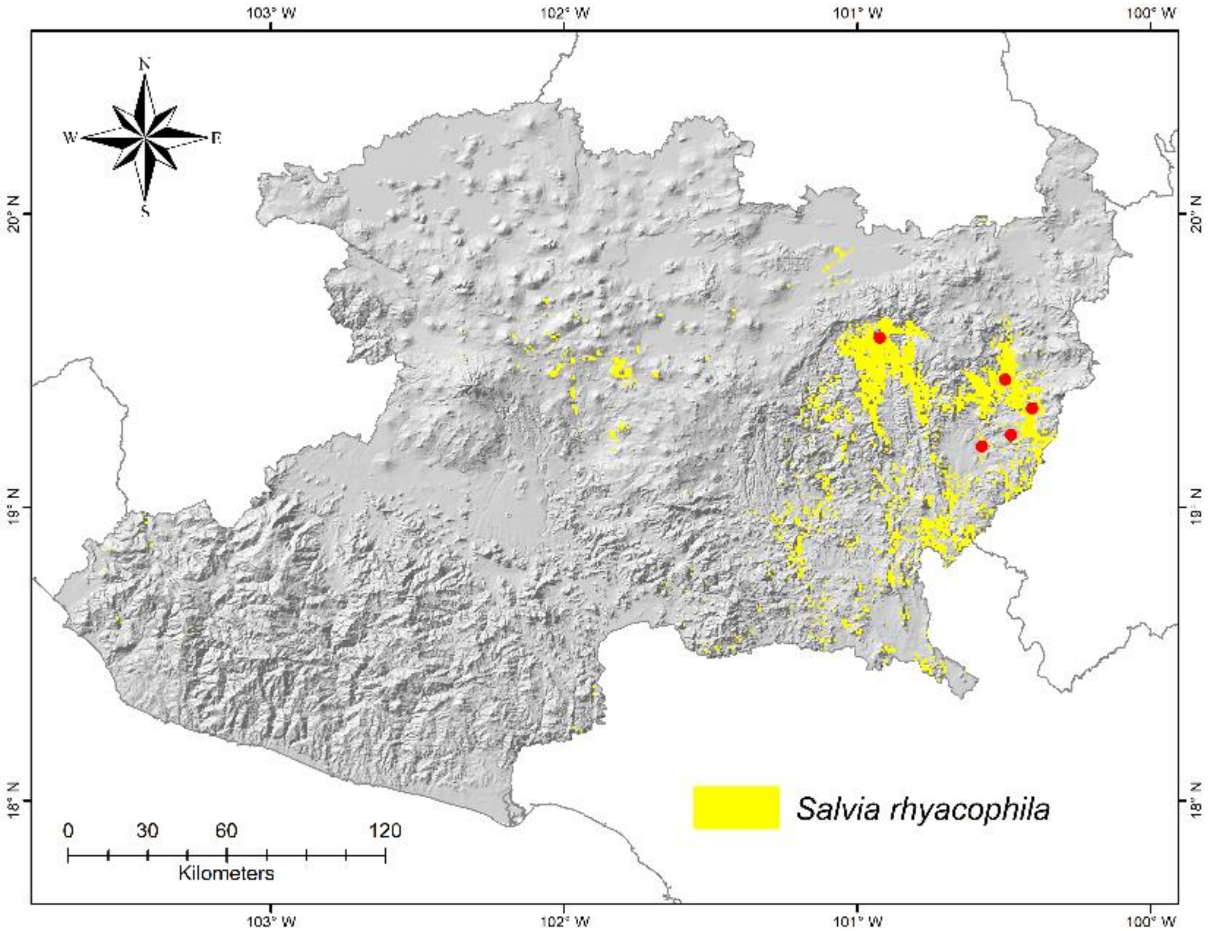
103° W
102° W
101° W
100° W
20° N
19° N
18° N
N
S
W
E
Salvia rhyacophila
0
30
60
120
Kilometers

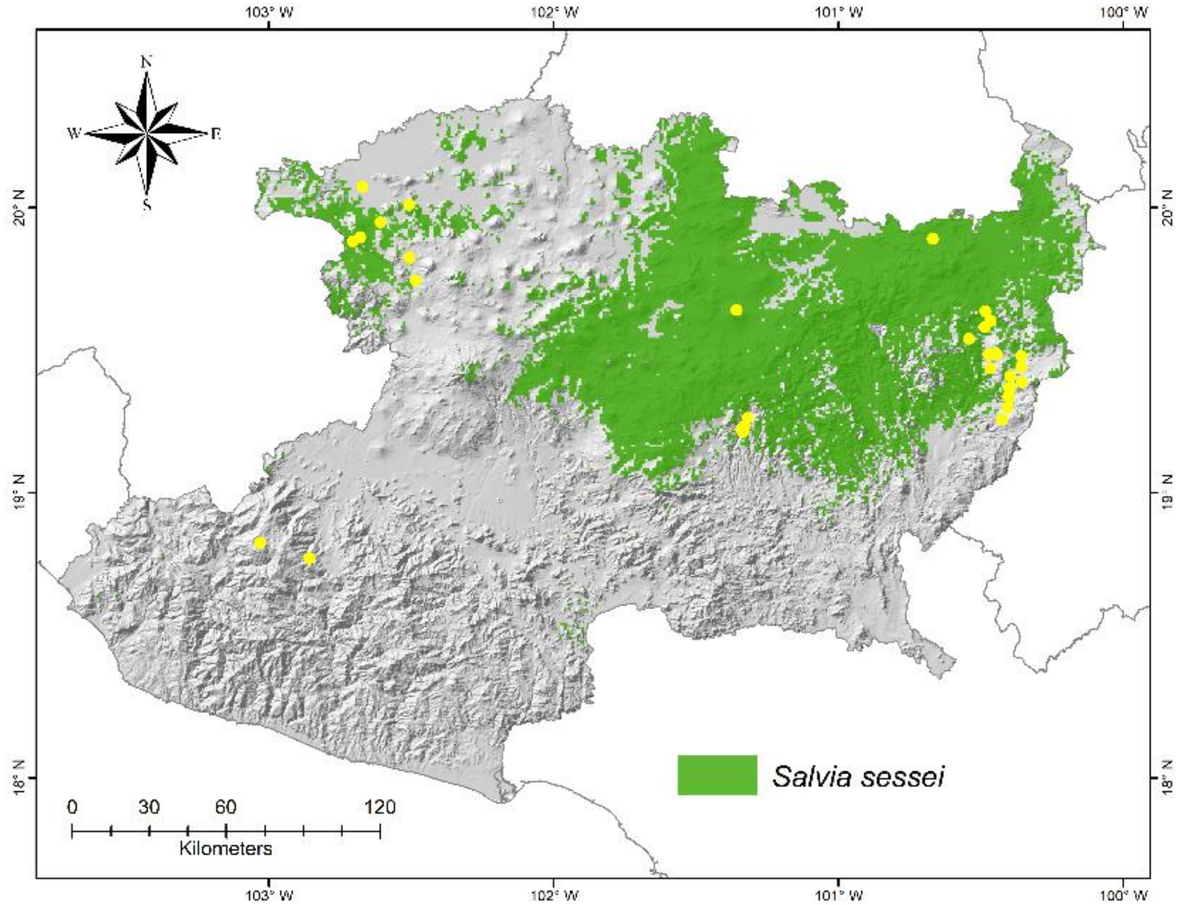
103° W
102° W
101° W
100° W
20° N
19° N
18° N
N
S
W
E
Salvia sessei
0
30
60
120
Kilometers

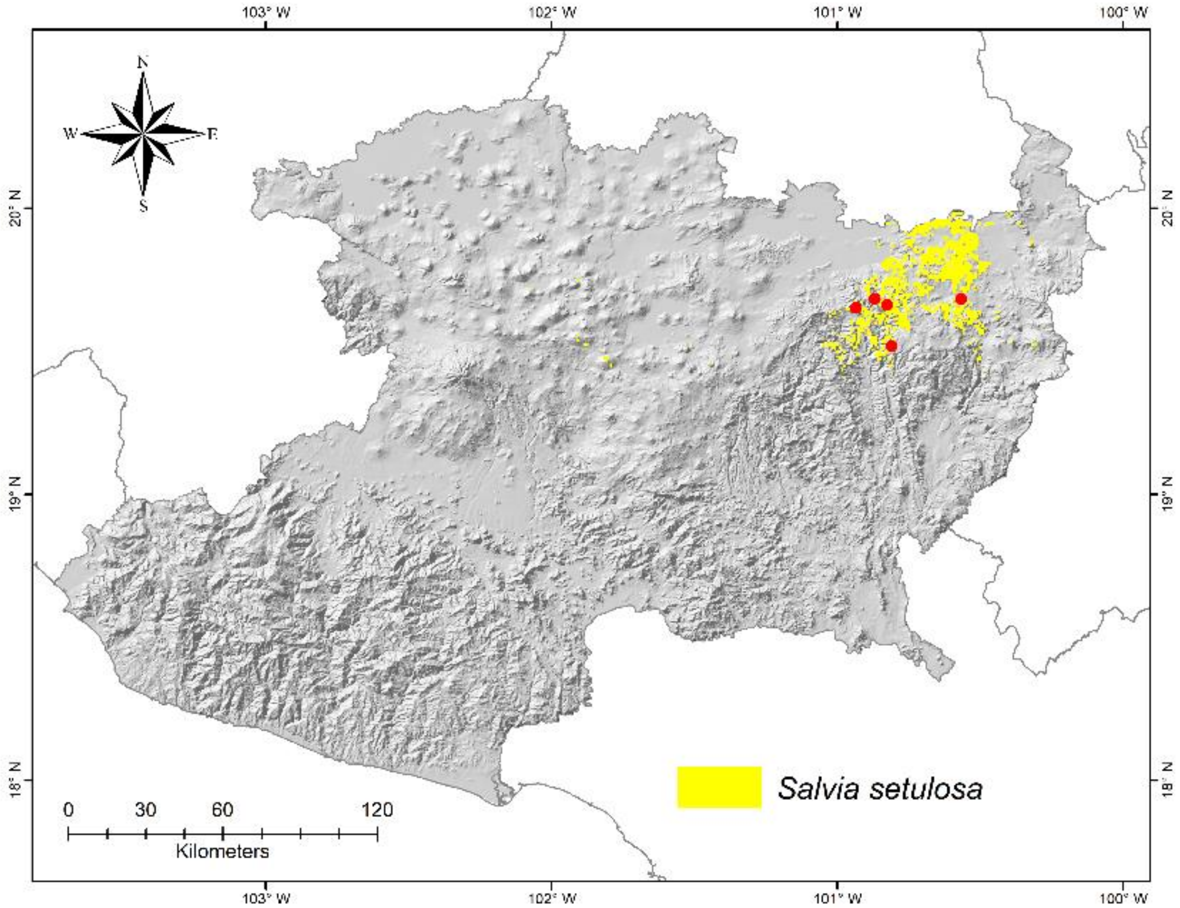
103° W
102° W
101° W
100° W
20° N
19° N
18° N
N
S
W
E
Salvia setulosa
0
30
60
120
Kilometers

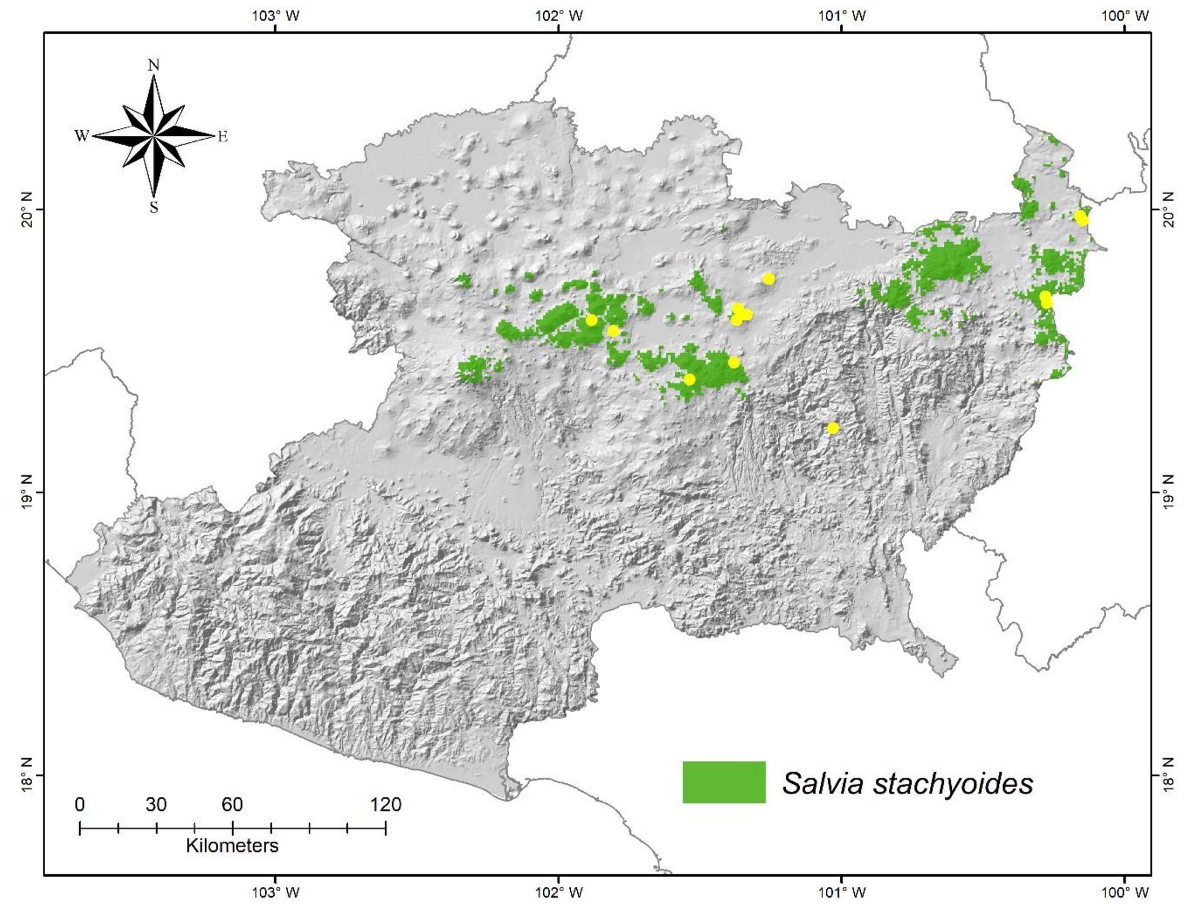

103° W
102° W
101° W
100° W
N
S
W
E
20° N
19° N
18° N
Salvia stachyoides
0
30
60
120
Kilometers

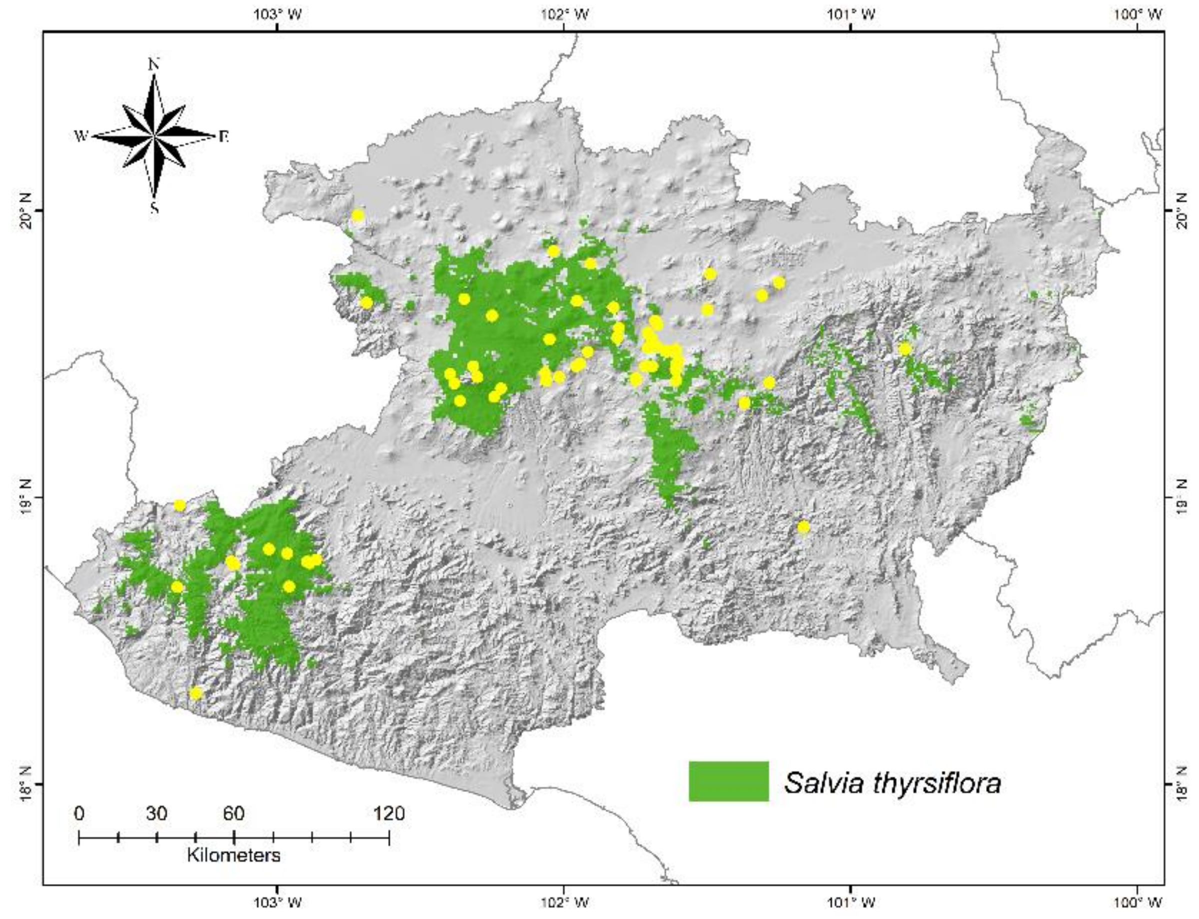

103° W
102° W
101° W
100° W
N
S
W
E
20° N
19° N
18° N
Salvia thyrsiflora
0
30
60
120
Kilometers

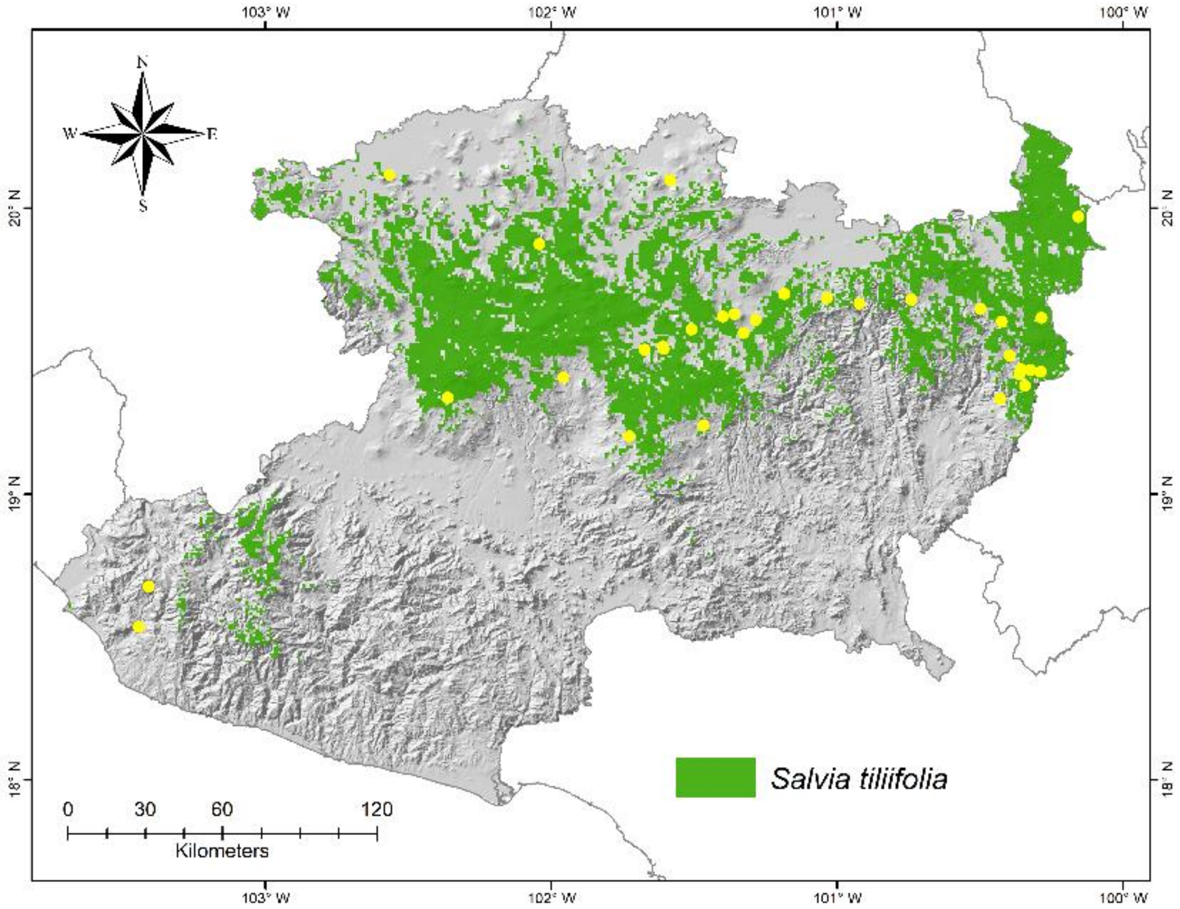
103° W
102° W
101° W
100° W
20° N
19° N
18° N
N
S
W
E
Salvia tiliifolia
0
30
60
120
Kilometers

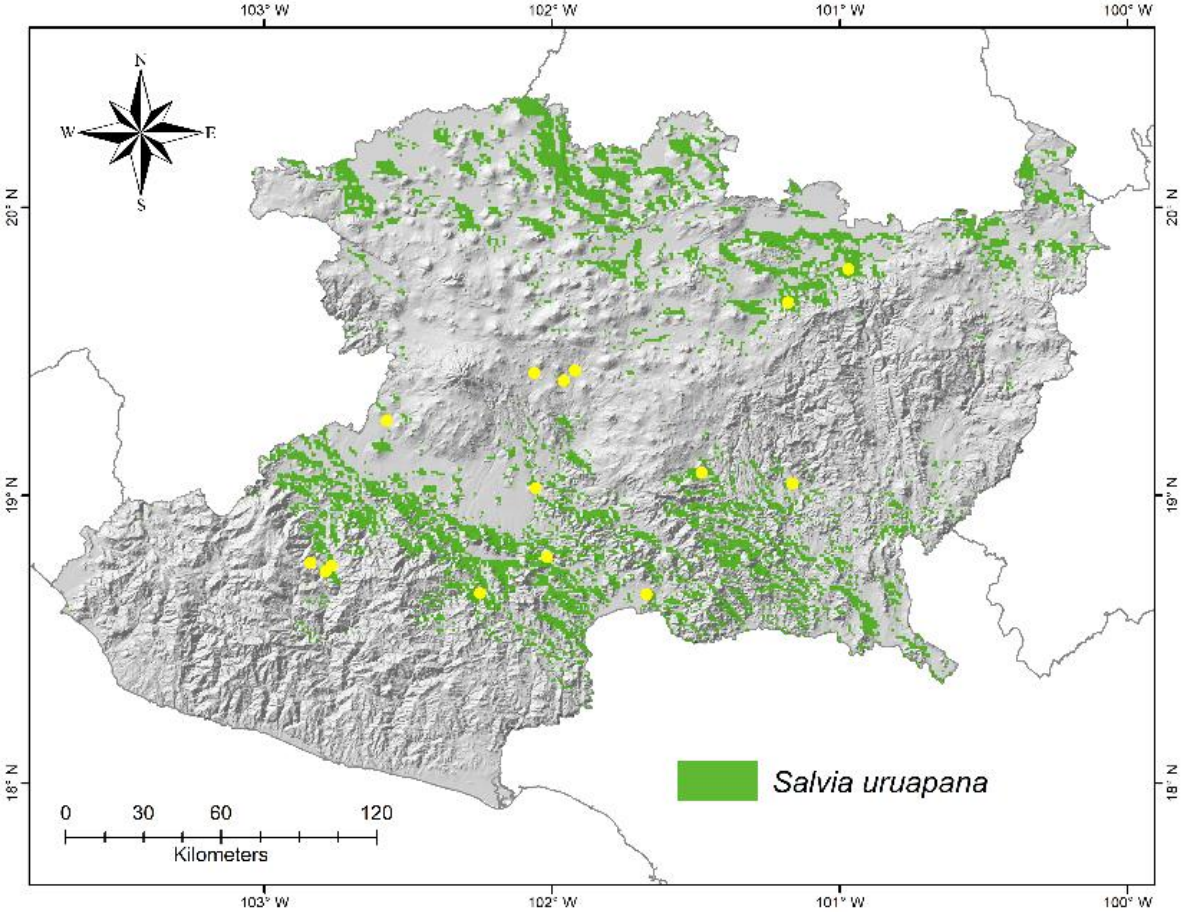
103° W
102° W
101° W
100° W
20° N
19° N
18° N
N
S
W
E
Salvia uruapana
0
30
60
120
Kilometers
