# Supplementary material for: Distribution, richness and conservation of the genus Salvia (Lamiaceae) in the State of Michoacán, Mexico
Source: Biodivers Data J. 2020 Oct 29;8:e56827. doi: 10.3897/BDJ.8.e56827 (PMC7644648; doi:10.3897/BDJ.8.e56827)
Supplement: Supplementary material 4 — Configuration of Maxent [file bdj-08-e56827-s004.doc]

**Supplementary file 4** –**Performance of ENMs selected based on AIC for each *Salvia* species.**

Evaluation metrics of Maxent ENMs generated with the ENMeval program for the three species of *Salvia* for which the statistical validation could be carried out. FC= Feature class (Logistic); MR= Multiple regularization; ΔAICc= Delta Akaike Information Criterion.

| Species | Number of records | FC | MR | ΔAICc |
| --- | --- | --- | --- | --- |
| *Salvia elegans* Vahl | 25 | L | 3 | 0.0 |
| *Salvia iodantha* Fernald | 22 | L | 2 | 0.0 |
| *Salvia mexicana* L. | 24 | L | 3 | 0.0 |
